# The Aluminum-Ion Battery: A Sustainable and Seminal Concept?

**DOI:** 10.3389/fchem.2019.00268

**Published:** 2019-05-01

**Authors:** Tilmann Leisegang, Falk Meutzner, Matthias Zschornak, Wolfram Münchgesang, Robert Schmid, Tina Nestler, Roman A. Eremin, Artem A. Kabanov, Vladislav A. Blatov, Dirk C. Meyer

**Affiliations:** ^1^Institute of Experimental Physics, TU Bergakademie Freiberg, Freiberg, Germany; ^2^Samara Center for Theoretical Materials Science, Samara State Technical University, Samara, Russia; ^3^Helmholtz-Zentrum Dresden Rossendorf, Institute of Ion Beam Physics and Materials Research, Dresden, Germany; ^4^Samara Center for Theoretical Materials Science, Samara University, Samara, Russia

**Keywords:** aluminum-ion battery, cathode, post-lithium, electrolyte, resources

## Abstract

The expansion of renewable energy and the growing number of electric vehicles and mobile devices are demanding improved and low-cost electrochemical energy storage. In order to meet the future needs for energy storage, novel material systems with high energy densities, readily available raw materials, and safety are required. Currently, lithium and lead mainly dominate the battery market, but apart from cobalt and phosphorous, lithium may show substantial supply challenges prospectively, as well. Therefore, the search for new chemistries will become increasingly important in the future, to diversify battery technologies. But which materials seem promising? Using a selection algorithm for the evaluation of suitable materials, the concept of a rechargeable, high-valent all-solid-state aluminum-ion battery appears promising, in which metallic aluminum is used as the negative electrode. On the one hand, this offers the advantage of a volumetric capacity four times higher (theoretically) compared to lithium analog. On the other hand, aluminum is the most abundant metal in the earth's crust. There is a mature industry and recycling infrastructure, making aluminum very cost efficient. This would make the aluminum-ion battery an important contribution to the energy transition process, which has already started globally. So far, it has not been possible to exploit this technological potential, as suitable positive electrodes and electrolyte materials are still lacking. The discovery of inorganic materials with high aluminum-ion mobility—usable as solid electrolytes or intercalation electrodes—is an innovative and required leap forward in the field of rechargeable high-valent ion batteries. In this review article, the constraints for a sustainable and seminal battery chemistry are described, and we present an assessment of the chemical elements in terms of negative electrodes, comprehensively motivate utilizing aluminum, categorize the aluminum battery field, critically review the existing positive electrodes and solid electrolytes, present a promising path for the accelerated development of novel materials and address problems of scientific communication in this field.

## Introduction

In 1900, Thomas A. Edison started developing a new battery for electronic vehicles. His final nickel-iron battery, patented in the USA in 1901 (Edison, 1901), became the most commercially successful product of his life^1^. It took him around 10 years, more than 50,000 experiments, and a withdrawal of the first version of the battery from the market before coming up with the right combination of materials to finally provide the best commercial battery available, as discussed by Dyer and Martin (2010): a lighter, more reliable, and up to three times more “powerful” battery than the existing lead-acid battery. At present, calls for another leapfrogging technology are becoming louder. Currently, the lithium-ion battery is the highest energy- and power-dense commercial product but there is demand for a new battery exhibiting an even higher energy density, better safety, and lower costs, especially for electric vehicles (EV), stationary storage, and mobile devices.

However, to develop a new battery chemistry today and especially in Europe, certain constraints need to be considered in order to obtain a sustainable, resource-efficient, and thus seminal concept. In this review article, we first describe the constraints of a sustainable and seminal battery chemistry. Subsequently, we present an assessment of the chemical elements in terms of negative electrodes and comprehensively motivate utilizing aluminum, but also indicate the challenges connected to it. In the second part, we categorize the aluminum battery field, define and introduce the aluminum-ion battery, and summarize the current state of knowledge. Finally, we critically review the existing positive electrodes and solid electrolytes and present a promising path for an accelerated development of novel materials. The review concludes by addressing problems of scientific communication in this field.

### Constraints for Novel Battery Chemistries

Over the next few years, the establishment of EV will be an important, if not the most important, driver for new battery chemistries^2^ alongside electric utility and public transport vehicles. The policies of Europe and in particular China with its huge car market and intense efforts (investments of billions of USD) reflect this (Lienert et al., 2019). The key to increasing the market acceptance of EV technologies are reducing costs and increasing the range under electric power, which will require continuous advances in battery chemistries (Muldoon et al., 2014). Additionally, the Norwegian Parliaments “Decision 672” obliges all shipping companies to only allow emission-free vehicles to enter the World Heritage waters of Norway from 2026 onwards^3^. This will also result in an increasing demand for energy storage in the marine sector, since other countries will follow suit. Power tools as well as medical tools and devices further contribute to the increasing need for energy storage^4^.

With the increase of renewable energy transformations from the sun, water, wind, and geothermal energy (in Germany around 36.2% of the electricity demand was generated from renewables in 2017^5^), the temporal storage of produced electric energy by “stationary storage” becomes necessary in order to intercept both under- or overdemand and under- or oversupply at different time scales from milliseconds to weeks and months. Furthermore, the storage can be used to dispatch large amounts of energy for frequency regulation, potentially preventing propagation of system disturbances, and providing additional flexibility for managing stability *in lieu* of demand response or load shedding (National Academies of Sciences, 2017). Since the amount of storable energy is directly proportional to the amount of active material, the cost per kWh is a driving factor of novel battery chemistries for these stationary storages, that will consume several orders of magnitude more raw materials.

The global demand for such energy storage is on the rise. In 2016, approximately 460 GWh of rechargeable electrochemical cells were produced worldwide (Pillot, 2017). An annual growth rate of about 8% overall and 25% for lithium-ion cells (in respect to revenues given in EUR) is expected. Besides the lead-acid technology for the use in car (SLI) batteries, the lithium-ion technology will also dominate the secondary storage market in the next decade due to its mature state. Predominantly, large electronic companies are pushing this technology forward, which is also reflected in the exponentially increasing number of patents. The lithium-ion battery is still the most attractive and best-commercialized battery, and target values of 150 USD/kWh will be realized soon, while its energy density has increased by almost a factor of four since its commercialization in 1991. The learning curve, however, is now flat and the physicochemical limit will soon be reached (Janek and Zeier, 2016; Thielmann, 2016). An important drawback of the lithium-ion system is the requirement of the aprotic (non-aqueous, organic) liquid electrolyte for ionic transfer (Schnell et al., 2018). Many of the issues these batteries face—safety concerns, capacity fading, aging, the cumbersome electrolyte filling and wetting process during production, and the extensive formation procedure—contribute to high costs and can be traced back to this liquid electrolyte (Schnell et al., 2018). Safety concerns, in fact, arise from the flammability of the solvents and there have been numerous incidents of burning batteries (Feng et al., 2018). It was therefore decided by the Governing Council of ICAO (International Civil Aviation Organization) to ban the transport of lithium-ion batteries as cargo in passenger aircrafts^6^.

For these reasons, new disruptive technologies with higher safety and higher theoretical energy density than existing lithium-ion batteries (Schnell et al., 2018), such as all-solid-state or high-valent^7^ batteries (Muldoon et al., 2014; Canepa et al., 2016; Schnell et al., 2018) are required. The roadmap for lithium-ion batteries shows that the use of lithium-metal negative electrodes inside all-solid-state batteries is the next important step envisioned for application after 2025 (Muldoon et al., 2014; Thielmann, 2017; Schnell et al., 2018), since it offers the potential for a dramatic improvement in energy density and safety. This all-solid-sate battery will become the benchmark for all upcoming battery concepts.

Tightly connected to a sustainable and seminal novel battery chemistry is the availability of (raw) materials and their best combination. Making use of earth-abundant metals as negative electrodes^8^ has become one of the hottest issues in the past years (Zhao et al., 2018), since companies as well as public authorities have become increasingly concerned about the supply risk of mineral resources. Numerous elements are needed for all kinds of utilization in building, manufacturing, and even the service sector, which may be in competition with the battery sector. Thus, resource-consuming industries face a number of risks regarding security of supply: the increase and volatility of prices are considered the most relevant risks (Bardt, 2016). The latter becomes increasingly important in particular, as cell costs decrease toward 100 USD/kWh (Olivetti et al., 2017,^9^).

Overall, our planet provides enough material resources. Crucial to their use, however, is how commodity prices develop, how accessible the markets are, how much energy is needed for mining and purification, and what environmental impact (CO_2_ and waste emissions, nature transformation, water demand, etc.) is expected in order to make these resources available for further processing. For the lithium-ion battery, for example, several analyses were done (see for instance Mohr et al., 2012; Olivetti et al., 2017). With the growing demand for lithium-ion batteries, the demand for lithium, cobalt, phosphorous, and other metals used within those batteries will also increase. While cobalt and phosphorous are already classed as “critical” by the EU^10^, Olivetti et al. (2017) pointed out that there may be challenges in rapidly scaling up the use of materials associated with lithium and cobalt in the short term.

Moreover, the development of a seminal novel battery chemistry also needs to be placed in a much broader context. In order to address the global challenges we face, the United Nations claims 17 “Sustainable Development Goals” as the blueprint to achieve a better and more sustainable future for all (United Nations, 2015). From a technical point of view, new sustainable technologies need to be developed, technologies and thus chemistries that take resource productivity and renewable/clean energies into account. For stationary storage in particular it must be considered that the production of batteries requires vast amounts of metals and other raw materials. Due to long battery lifetimes and multiple end uses, these metals are then sequestered for several decades and cannot immediately be recycled to provide significant short-term supply (Vidal et al., 2013; Olivetti et al., 2017). Furthermore, for mining deposits with lower concentrations of these respective metals, the expendable energy becomes a limiting factor (more than 10% of world energy consumption is already used for extraction and processing of mineral resources; Vidal et al., 2013) and poses an important risk in the battery market. *Conditio sine qua non* for the battery producing and applying industry to exist and continue sustainably is thus exploiting the deposits in an environment-friendly and energy-reducing way.

Sustainable and seminal battery chemistries thus need to be developed under these indicated constraints of the end user, societal, and industrial demands, policies and raw material supply. Certainly, any battery chemistry can contribute to a diversification of the battery market. If a novel battery chemistry exhibits a promising performance, its industrial up-scaling will be “measured” against the indicated challenges. Therefore, high-abundant chemical elements like aluminum should be the focus of battery chemistry research. In the following section, the chemical elements of the periodic table have been assessed under these constraints.

## Concept Assessment

This chapter is based on the articles (Meutzner et al., 2018a; Schmid et al., 2018; Nestler et al., 2019b), and envisions an all-solid-state battery with a metallic negative electrode. For the conceptual development of resource-, environmental-, and cost-optimized novel electrochemical energy storage, an evaluation system has been worked out that ranges from the potential material for the storage concept to its application.

The chemical elements used for the basic components of a battery (negative electrode, electrolyte, positive electrode) are a basic distinguishing feature and these form the core of each concept. The first step in assessing and selecting the elements of the periodic system up to the number 94, is to ensure their suitability for sustainable and seminal battery chemistries. There are different ways of rating a material of which the “Criticality Assessments” by the EU^10^ or the “Resource Risk Index” *RRI* (Bardt, 2016, 2017) are two possibilities. The EU rates, among other, magnesium, phosphorous, tungsten, and cobalt as critical. The *RRI* indicates the current resource risk for the German industry, whereby all elements with an *RRI* from 16 to 25, such as tungsten (20.4), tin (17.8), magnesium (15.6), and cobalt (16.3) have to be considered critical. Phosphorous (14.3), lithium (12.3), aluminum (12.0), and lead (9.5) currently have a medium to low risk.

**Figure 1:**
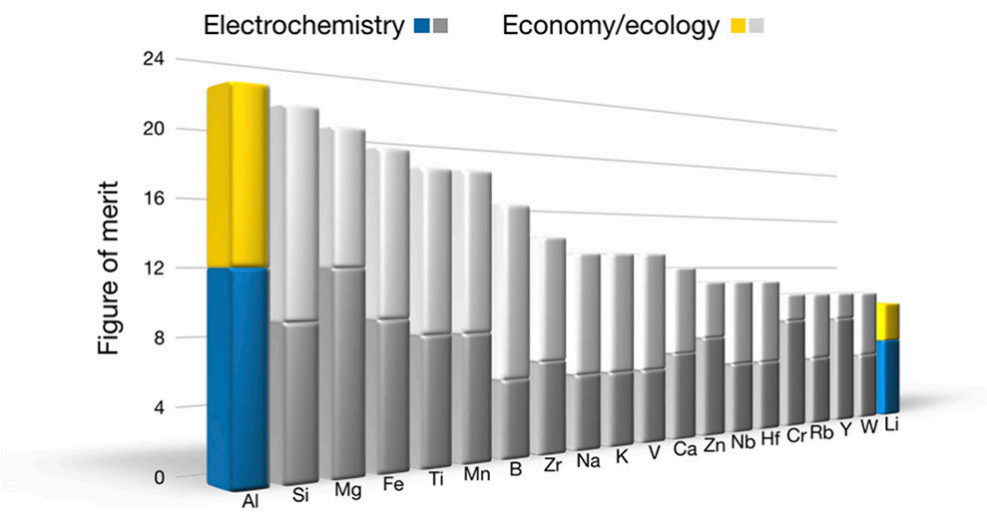
Result of the assessment of the elements up to the number 94. The highest possible value is 22. Light colors are related to economic and ecologic, whereas dark colors are related to electrochemical aspects. Aluminum is ranked first as negative electrode for an all-solid-sate aluminum-ion battery.

The parameters considered for assessing the elements can be distinguished very broadly into two categories—electrochemistry and economy—and the rating can be adjusted for the task at hand (see Schmid et al., 2018). Here, the negative electrode is chosen: When we assume an all-solid-state battery based on oxygen-containing compounds (assuming a design and values given by Schnell et al. (2018), the solid electrolyte Li_7_La_3_Zr_2_O_12_, and the positive electrode consisting of 70 vol.-% LiNi_0.8_Co_0.15_Al_0.05_O_2_ and 30 vol.-% Li_7_La_3_Zr_2_O_12_), the element with the largest share besides oxygen with 46 at.-% is the mobile species (and thus the negative electrode) with 33 at.-%, since it is found in the negative electrode, the electrolyte, and the positive electrode. All other elements amount to 21 at.-%. For the rating, each parameter has been evaluated with a score. Adding up all scores, the element with the highest number is the most promising for future uses. According to this algorithm, aluminum is the highest-potential candidate for a battery chemistry based on a metal-negative electrode (Figure 1), congruent with Zhao et al. (2018).

Currently, besides the trivalent aluminum ion, the alkali metals such as sodium and potassium (Elia et al., 2016) and several other mobile ions such as bivalent calcium and magnesium are of high relevance for secondary post-lithium high-valent ion batteries (Nestler et al., 2019a). A recent review by Canepa et al. (2016) states that most of the research on high-valent ions is done on non-aqueous magnesium (ca. 81%), 15% with zinc, 3% with calcium, and 1% with others including aluminum (as well as strontium and barium). The use of high-valent ions increases the number of electrons involved in the electrochemical process and thus, in principle, leads to high capacity values. This is considered by the algorithm used.

**Figure 2:**
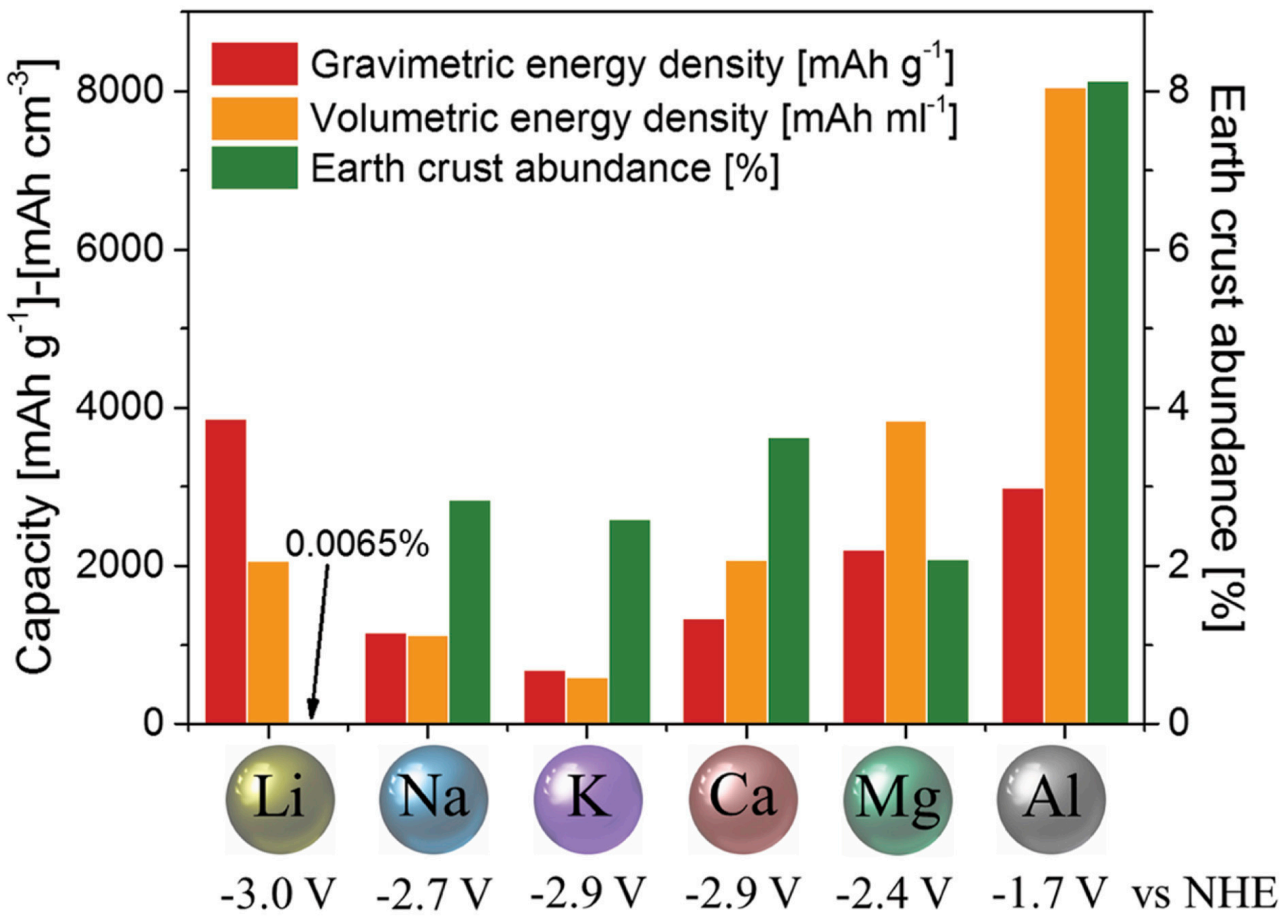
Comparison between gravimetric and volumetric capacities, standard reduction potential and earth's crust abundance of metal negative electrodes used or proposed for application in electrochemical storage systems (Fleischer, 1953; U. S. Geological Survey, 2015). Figure reproduced with permission from Elia et al. (2016) © 2016 WILEY-VCH Verlag GmbH & Co. KGaA, Weinheim.

Based on the assessment, we suggest that the concept of an aluminum-based (high-valent ion) rechargeable all-solid-state battery appears highly promising for meeting future demands. Here, aluminum metal is used as the negative electrode with the ability to exchange three electrons during the electrochemical process (Al → Al^3+^ + 3e^−^). Aluminum, in fact, possesses one of the highest theoretical volumetric capacities of all elements. A very convincing representation for the utilization of aluminum as a negative electrode in an aluminum-based battery was already provided by Muldoon et al. (2014) and Elia et al. (2016) (Figure 2). Accordingly, it offers the great potential of a volumetric capacity four times higher compared to lithium (8.0 vs. 2.0 Ah/cm^3^), while gravimetric capacities are comparable (3.0 vs. 3.8 mAh/g). The energy density (volumetric capacity times usable voltage) of a battery pack becomes important when there is a limited volume for mounting. A high energy density is therefore more desirable for mobile devices (such as EV, assistive robots, or power tools). The more portable the device (such as personal electronics), the less space is available for its battery and the energy density plays a crucial role (Muldoon et al., 2014). With the same volume of a battery based on aluminum-metal negative electrode, a car would potentially have two to six times the range compared to commercial lithium-ion batteries (assuming a liquid-electrolyte-type as well as an all-solid-state-type lithium-ion battery with operating voltages of 3 V as well as an aluminum-ion all-solid-state-type battery with 1.7 V).

It has to be noted that an aluminum-metal negative electrode has a less negative standard reduction potential compared to other interesting candidates (Figure 2) pointing at a possible limitation in first approximation. A more careful look at aluminum will exhibit encouraging features for its use, however, it will also exhibit challenges as discussed in the next chapter.

## Aluminum

### Raw Material, Abundance, Resources, Production, and Recycling

The high abundance of aluminum of around 8 wt.-%, renders it the most abundant metal in the earth's crust and the third most abundant element after oxygen and silicon (cf. Figure 2). According to Bardt (2017) and the EU^10^, aluminum is not rated as critical in the reserves-to-production ratio, in the political, and in the supply risks because it comes from various different sources. Due to its ignoble character, it occurs only rarely as a native metal, in the form of aluminum silicates. Thus, pure aluminum needs to be produced from minerals or by recycling from scrap. Bauxite is the most important mineral for the production of primary aluminum: 1 kg of aluminum can be obtained from 4 kg of bauxite (Habashi, 2003; Ostojic et al., 2014). The concentrations of aluminum in the respective raw material is more than two orders of a magnitude higher in comparison to lithium, which is of importance with regard to the moved amount of soil for mining. In regards of at.-%—the parameter of choice for battery use—still, more than twice of aluminum atoms can be mined from 1 kg of raw material compared to lithium. This is another advantage of utilizing aluminum as a metal-negative electrode in batteries.

Every continent has its own mining, production, and recycling sites. The latter is particularly important: In order to significantly decrease the energy demand of aluminum production, a recycling infrastructure was established, early on. Regarding the bond dissociation energies Δ_f_*H*^298^ for the Al–O as well as for the Al–Al bond, 512(4) vs. 186(9) kJ/mol (Dean, 1999), respectively, it becomes understandable that the energy consumption of the total aluminum production process from aluminum scrap can be reduced by 95% (Habashi, 2003). Hence, today, 35% of the aluminum demand is supplied through recycled, secondary aluminum^11^.

The aluminum industry, however, is responsible for around 1% of man-made greenhouse gas emissions, with around 40% resulting from the aluminum production process itself (direct emissions) and around 60% resulting from electricity power generation (indirect emissions) for obtaining the aluminum metal (International Aluminium Institute, 2009). Around 1 kg of aluminum generates (5–40) kg emissions of CO_2_. Therefore, a systemic thinking is important for an increase in aluminum production and for any aluminum-containing applications, taking renewable energy into account in order to reduce emissions and consumption of non-renewable energy carriers. Here, the aluminum production could be seen as one step in an aluminum-ion battery value-added chain: Storage and transport of electric energy via aluminum-metal from the place of production (hydro-electric power plants, wind or photovoltaic parks) to the place of its usage. Due to its high demand in electrical energy, most production plants are situated next to (hydro-electric) power stations. To produce 1 kg of aluminum, temperatures of around 1,000°C as well as (9–12) kWh of electrical energy are necessary, with process efficiencies of (85–95)% (Habashi, 2003). Around 5 kWh per kg could theoretically be retrieved in an aluminum-ion battery (cf. Table 1).

**Table 1:** Comparison of characteristics of aluminum with lithium, the benchmark element (the bold marked numbers indicate advantageous values).

	**Aluminum**	**Lithium**
Abundance (wt.-%)^a^	**~8.3⋅10^−2^**	~1.7⋅10^−5^
Criticality *RRI* (Bardt, 2017)	**12.0**	12.3
Criticality by EU (supply risk/economic importance)^10^	**0.5**/6.5	1.0/**2.4**
Energy for production of 1 kg (kWh) (Mahi et al., 1986; Kipouros and Sadoway, 1998; Habashi, 2003)	**9–12**	32–40
Ionic radius Al^3+^/Li^+^ (pm) (Shannon, 1976)	**39**	59
Mass density (kg/m^3^) (Haynes, 2011)	2,699	**534**
Mean distribution in earth crust (g/t)^b^	**~83**	~0.17
Melting point of metal (°C) (Haynes, 2011)	660.32	**180.50**
Melting point of oxide (°C) (Haynes, 2011)	2,054(6)	**1,570**
Price (USD/kg)^*^	**2**	300
Recycling ratio worldwide (%) (United Nations, 2011)	**50–70**	< 1
Reduction potential (V) vs. SHE (Haynes, 2011)	−1.676	**−3.040**
Reserves (Mt) (Olivetti et al., 2017; U. S. Geological Survey, 2018)	**7,500**	13–40
Resources (Mt) (Olivetti et al., 2017; U. S. Geological Survey, 2018)	**18,750**	33–64
Theoretical specific energy (kWh/kg)^**^	4.95	**11.64**
Theoretical energy density (Wh/cm^3^)^**^	**13.36**	6.20
Theoretical gravimetric capacity (kAh/kg)	2.98	**3.83**
Theoretical volumetric capacity (Ah/cm^3^)	**8.05**	2.04
Toxicity (Holleman and Wiberg, 2007)	**(no)**	low

a *https://www.chemicool.com/elements/lithium.html (December 2, 2018)*.

b *https://en.wikipedia.org/wiki/Abundances_of_the_elements_(data_page) (December 2, 2018)*.

* *The prices have been estimated utilizing values given by various suppliers of raw materials as well as by various price reports*.

** *Obtained by multiplying 1.66 V (aluminum) and 3.04 V (lithium) to the respective capacities*.

It should be noted, that for the production of lithium from minerals, temperatures of up to 1,150°C are applied (Tran and Luong, 2015; Schmidt, 2017). Subsequently, metallic lithium is, like aluminum, also produced by fused-salt electrolysis in an electrolytic cell using lithium chloride^12^ at temperatures of the order of 500°C (Mahi et al., 1986; Kipouros and Sadoway, 1998) and with an electrical energy consumption of (32–40) kWh per kg. Furthermore, similar environmental issues, such as the formation of unwanted gases and their volumes, occur during the electrolysis processes of both aluminum and lithium. Precise calculations of greenhouse gas emissions for the lithium metal production seem to be lacking thus far.

Taking these given numbers into account, the electrical energy spent is roughly three to five times lower for aluminum in comparison to lithium providing the same theoretical gravimetric or volumetric capacity of a metal-negative electrode in a battery.

### Bulk and Surface Properties, Corrosion

Aluminum crystallizes in the space group $Fm\bar{3}m$ with room-temperature lattice parameter *a* = 4.04950(12) (Witt, 1967), that is stable up to its melting point (Hatch, 1984). According to FIZ Karlsruhe GmbH (2018) no other aluminum structure is known; however, a structural phase transition was induced by intense laser radiation (Guo et al., 2000). This structural stability makes aluminum an interesting negative electrode. So far, more than 18,000 of roughly 194,000 entries of aluminum-based compounds with two and more constituents are listed in the Inorganic Crystal Structure Database (FIZ Karlsruhe GmbH, 2018). This provides a large amount of crystal structures to develop design principles for aluminum-ion conductors. Most of the metallic elements readily alloy with aluminum and a wide variety of intermetallic phases can be formed (Hatch, 1984). This is important for tuning the electrochemical properties of aluminum as a battery component. Aluminum is considered to approach an “ideal” metal or a free electron gas (Nakashima et al., 2011). The three valence electrons contributing to the free electron gas give aluminum an excellent electrical conductivity of 37.7 MS/cm (resistivity of 26.5 nΩcm), which is approximately 65% that of copper (Hatch, 1984). Given the respective electrical resistances of aluminum and copper, for the same electrical resistance of a conductor, the aluminum conductor has a 30% lower weight in comparison to copper. This also makes aluminum an ideal candidate for current collectors already in use (Myung et al., 2011). Accordingly, the thermal conductivity of aluminum with 237 W/mK approaches 59% that of copper (Hatch, 1984), which also makes aluminum an excellent part of the battery to transfer or distribute the heat, due to the charge or discharge of a battery.

With regards to its wide use, as well as the development of new applications, the debate on whether aluminum is harmless or not is still ongoing. Up to now, there is still a lack of adequate data of risk assessment, implying that there is no evidence that aluminum is harmful to the human body, neither due to dermal penetration nor by ingestion (World Health Organization, 1997; Bundesinstitut für Risikobewertung, 2014; European Commission, 2014). Lithium, in contrast, when exposed to air reacts to acidic lithium hydroxide and is supposed to be toxic when ingested (Aral and Vecchio-Sadus, 2008). Tkatcheva et al. (2015) reported that lithium can be accumulated in the brain tissue, which potentially explains its action as a mood stabilizer^13^. However, detailed studies are unavailable due to the lack of regulations for lithium (Tkatcheva et al., 2015). This makes aluminum an even more interesting material for batteries. It has to be noted that compounds made from aluminum or lithium might have other toxicities.

One of the greatest challenges, connected to the use of aluminum as an active battery material, is its affinity to oxygen and thus the oxidation of the nascent aluminum surface that is exposed to oxygen, water, or another oxidant (Hatch, 1984; Vargel, 2004). The enthalpy of formation Δ_f_*H*^0^ of a solid oxide at standard conditions

(1) $$\begin{array}{r} 2\text{Al}+3/2{\text{O}}_{2}\to \text{A}{\text{l}}_{2}{\text{O}}_{3}, \end{array}$$

is −1,675 kJ/mol, which is higher than for the oxidation reaction of iron to Fe_3_O_4_ (−1,121 kJ/mol) (for Li_2_O it is −599.1 kJ/mol) (Linstrom and Mallard, 2017). At standard conditions, aluminum will thus instantaneously (<1 ms) form a very thin uniform and continuous amorphous surface layer of Al_2_O_3_ of the order of (2–10) nm thickness (Vargel, 2004). The film thickness triples in the presence of water vapor but the rate of formation is independent of the oxygen partial pressure (Hatch, 1984). This dielectric as well as amphoteric oxide layer behaves liquid-like under stress and therefore has self-healing abilities (Yang et al., 2018). The oxide layer is in compression with respect to the underlying aluminum and can sustain deformation without breaking (Vargel, 2004). Generally, the oxide film is stable over a *pH* range of about 4.0 to 8.6 (Deltombe and Pourbaix, 1958; Hatch, 1984).

On the one hand, this provides excellent resistance against the environment, e.g., oxidizing media such as air, water, etc., which is advantageous when transporting and handling aluminum (metallic lithium will face more pronounced corrosion problems during processing and delivering to manufacturing sites). Moreover, this is advantageous when aluminum is used as a current collector for (lithium-ion) batteries (Myung et al., 2011). The very thin oxide layer does not prevent electric conduction but adds a contact resistance of 35 kΩ (Oh et al., 1999). The tunneling effect realizes the electric conduction (Nakai and Miyazaki, 1964). On the other hand, this provides a barrier for solvation of aluminum (see bond dissociation energies above), that poses a challenge for the use of aluminum metal as a negative electrode. Thus, this natural oxide influences the electrochemical behavior (overpotentials) of aluminum. A second layer is generally formed on top of the oxide layer by a reaction with the adjacent gases and liquids; the continuous oxide layer closest to the metal surface changes to a hydroxylated film at the solid/gas interface (Hatch, 1984).

The behavior of aluminum in oxidizing environments can be visualized as a first approximation in a so-called Pourbaix diagram (Deltombe and Pourbaix, 1958; Vargel, 2004). In electrochemistry, a Pourbaix diagram is also known as a potential (*E*_H_)–*pH* diagram and can be seen as an equivalent of the well-known phase diagrams. It is calculated by the Nernst equation. The diagram shows possible stable (equilibrium) phases, e.g., in the aluminum-water system (Vargel, 2004; Ashby and Jones, 2012). There are conditions under which aluminum does not corrode (“immunity”) because there is no or a negative voltage driving force. Aluminum may corrode (“corrosion”) because there is a voltage driving force which hinders the formation of a stable oxide film on the surface. As a third possibility, aluminum may not corrode (“passivation”) because, although there is a voltage driving force, a stable oxide film forms on the surface (this may or may not be an effective barrier to corrosion).

**Figure 3:**
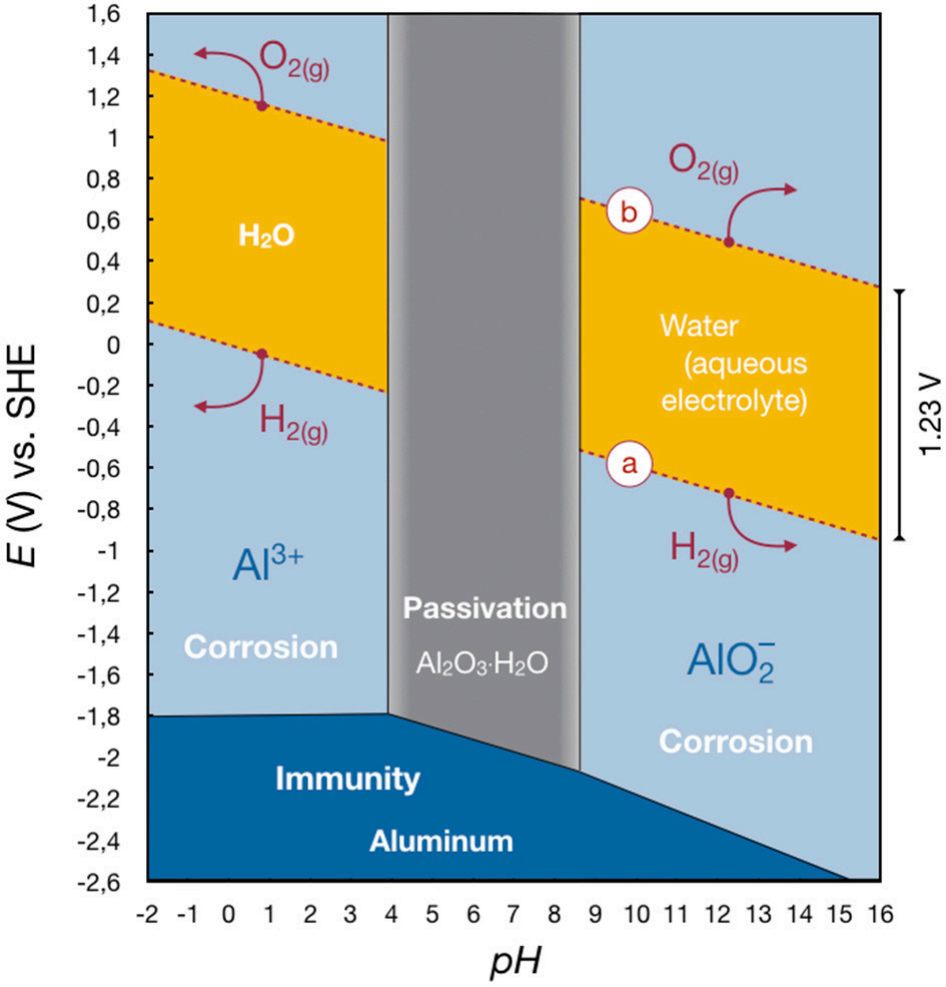
Pourbaix diagram of aluminum in water at 25°C showing its corrosion behavior. It depicts the basic oxidation/reduction reactions for aluminum in aqueous systems. Outside the yellow region, water breaks down, not the metal. It can be seen that a secondary aluminum-ion battery with an aluminum metal as negative electrode based on an aqueous system will not be possible since the aluminum cannot be plated both at low and high *pH*. It cannot be solved in a medium *pH*, as well. Therefore, just primary battery systems can be realized (cf. section Aqueous or Primary Aluminum Battery). Redrawn from Deltombe and Pourbaix (1958), Vargel (2004), and Ashby and Jones (2012).

At first, we want to consider pure water, with a stability region limited by the dashed lines in Figure 3. Outside this area, water is unstable and decomposes. Above line (b), oxygen is evolved in accordance with the reaction

(2) $$\begin{array}{r} 6{\text{H}}_{2}\text{O}\to 4{\text{H}}_{3}{\text{O}}^{+}+{\text{O}}_{2\left(\text{g}\right)}+4{\text{e}}^{-}, \end{array}$$

below line (a), hydrogen evolves in accordance with the reaction

(3) $$\begin{array}{r} 2{\text{H}}_{2}\text{O}+2{\text{e}}^{-}\to {\text{H}}_{2\left(\text{g}\right)}+2\text{O}{\text{H}}^{-}. \end{array}$$

If aluminum is present, the Pourbaix diagram becomes more complex. At highly acidic and highly alkaline conditions, respectively, an intrinsic gas evolution occurs on the surface until the aluminum or the water is consumed while dissolving Al^3+^ or ${\text{AlO}}_{2}^{-}$. This renders the amphoteric nature of aluminum. Between a *pH* of 4 and 8.6, hydrargillite Al_2_O_3_ · H_2_O is the stable phase and a passivation layer forms, protecting the aluminum. Indeed, this film is considered to be responsible for successful use of aluminum in many applications where a passivation is useful (e.g., as current collectors in liquid-electrolyte batteries). If the potential is sufficiently low, aluminum itself is immune to corrosion. The corrosion rate of aluminum in pure water is extremely low, even though the driving force for corrosion is very large (>+2.8 V) (Ashby and Jones, 2012).

**Figure 4:**
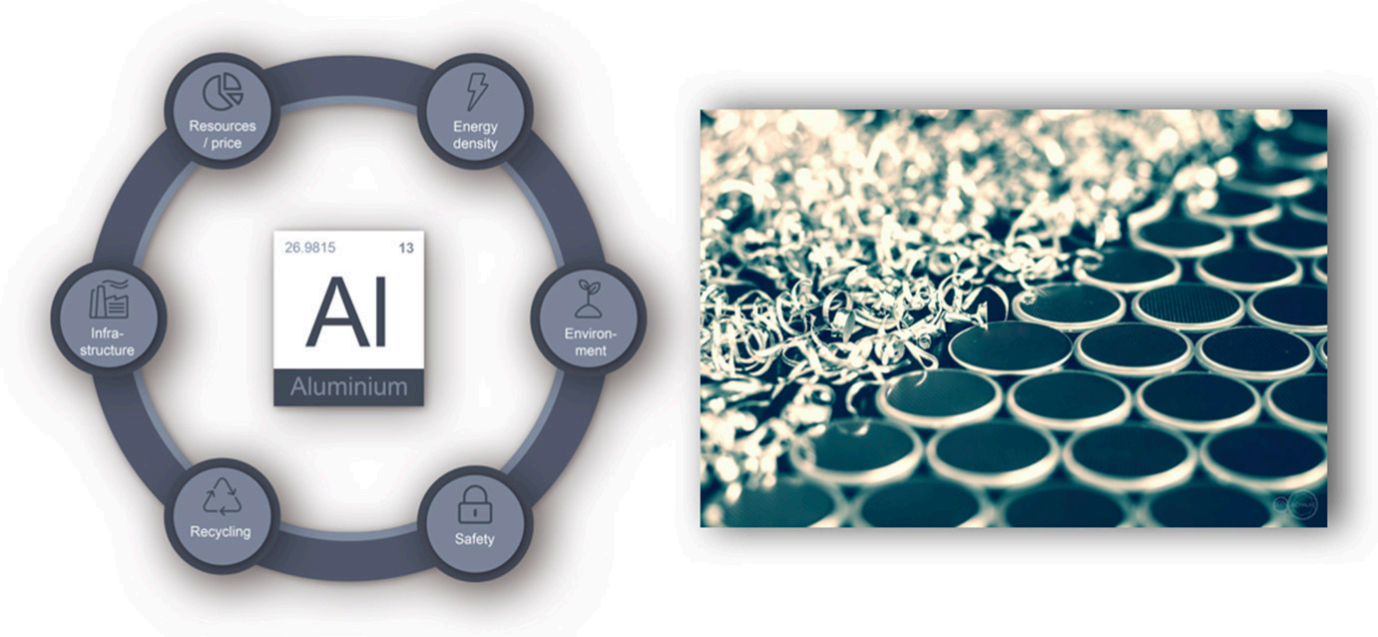
Advantages of utilizing aluminum as battery material (negative electrode, current collector, housing).

Aluminum as negative electrode bears several advantages compared to lithium, as well as weaknesses, which are summarized in Tables 1, 2 and Figure 4. Additionally, it is the world's most-used metal without iron contamination, easy to work with, in cold or hot conditions, and offers easy handling in ambient environment. Therefore, the marketing of aluminum battery technology is expected to become easier in comparison to the lithium battery technology (Zhang et al., 2018).

**Table 2:** Strengths and challenges of aluminum as negative electrode in a battery (in comparison to lithium).

**Strengths**	**Challenges**
•**Energy density**: Volumetric capacity is four times higher than for lithium. •**Environment**: Not harmful, environmentally benign, less electrical energy necessary for producing metallic aluminum (as negative electrode), less soil needs to be moved during mining due to higher concentrations. •**Infrastructure**: Matured and already well-established production, electroplating, manufacturing, recycling and scrap collecting infrastructures. •**Recycling**: Already established recycling technologies and plants. •**Resources/prize**: Small political supply risk, distribution of production and recycling plants all over the world, small price due to very high abundance. •**Safety**: Does not ignite in air, which can ensure greater safety of corresponding cells and ease of processing.	•**Electrochemical window**: Difficult to find liquid electrolytes, which enable dissolution and plating of elemental aluminum. •**Coulomb interaction**: High-valence state of +3 may introduce slow intercalation/deintercalation kinetics and ion conduction. •**Oxide layer:** Hindering of dissolution; redox potential may become more positive. •**Redox potential**: With −1.67 V rather low compared to lithium. •**High affinity to oxygen**: Influence on environmental conditions and small changes in the local chemistry. •**Research efforts**: Not intense; in the beginning, 30 years delay in comparison to lithium.

## Aluminum-ion Battery

**Figure 5:**
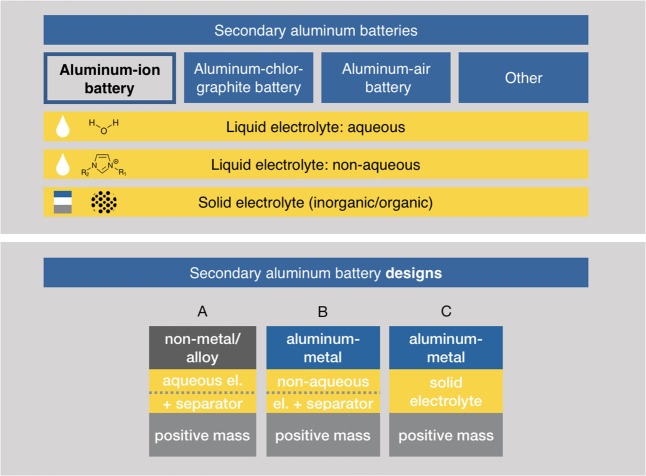
Categorization of aluminum batteries in regard to their operating scheme and their used type of electrolyte. Other battery types are dual-ion batteries (Zhao et al., 2018). Below, different conceivable secondary aluminum-ion battery designs are depicted. **(A)** This design does not make use of the full potential of aluminum since the negative electrode is either an alloy or a non-metal. The designs in **(B,C)** use aluminum metal as negative electrode, **(B)** uses a liquid and **(C)** a solid electrolyte.

In the literature, the term “aluminum-ion battery” is used for a variety of systems applying aluminum. Currently, a clear categorization is missing in regard to the, to this point, lacking research activities in this field (see below). We suggest a categorization as depicted in Figure 5. Strictly speaking, the aluminum-ion battery is just a subset of all variants published so far. As pointed out by Kravchyk et al. (2017), an “aluminum-ion battery” is characterized by the unidirectional flow of Al^3+^ ions from one electrode to another. In the following section, we restrict the term “aluminum-ion battery” to exactly those systems. Taking literature published on this point into account, four types of a secondary aluminum battery and three aluminum-ion battery designs can be identified depicted schematically also in Figure 5.

In order to exploit the high theoretical energy densities of an aluminum-ion battery (13.36 Wh/cm^3^, which is 1.6 times higher than gasoline^14^ of 8.6 Wh/cm^3^), a metallic negative electrode made of pure aluminum needs to be utilized. For this purpose, a stable electrolyte in regard to the electrochemical stability window is also demanded. A solid electrolyte could solve most of the issues connected to the disadvantages of highly corrosive or unstable liquid electrolytes. Finally, a positive electrode needs to be identified, which enables high capacities, high voltages, and thus high energy densities. For both the positive electrode and the (solid) electrolyte, ion conduction is of main importance. When a novel material exhibits a sufficient ion-conductivity the electronic conduction decides, whether such a material is used as a solid electrolyte (electronic insulator) or as a positive electrode (electronic conductor). It should be noted that an electronically insulating material could be transformed to a positive electrode, if, e.g., mixed with graphite or black carbon, and a redox reaction can take place at a constituting element and an electrical conductor could be doped to decrease electrical conductivity.

Therefore, one of the main tasks in this still early state of research should first be to identify materials with a sufficient ion conductivity comprising of non-critical elements. In the following section, the emphasis is set on these materials.

### Aluminum Battery Developments

In 2017, the TechVision Division of Frost Sullivan (2017) announced the aluminum-ion battery as one of the potential post-lithium battery systems for the first time. The average global annual growth of patent filing from 2010 to 2016 was around 29%. Patent filings for aluminum batteries started only in 2013. The top patent assignee is China. South Korea, North America, and increasingly, Europe are also becoming important actors. However, in Europe, the research activities are low. Apart from the ALION^15^ project, this type of battery is not part of the technological focus of the European Commission, for now. So far, there are no companies or startups directly involved in this battery technology, which indicates that this battery is still in its early stages. Research on aluminum batteries has become more extensive in the last 5 to 10 years. While until 2016 around 66 scientific articles regarding the aluminum-ion battery were published, in 2017 there were 55 articles^16^ (for comparison, around 8,000 articles were published in 2017 for the lithium-ion battery, alone). Most articles were from China (53%) and the United States (15%), whereas all other countries were below 8%. Most of the research is centered on ionic liquids and intercalation materials (positive electrodes), some dealing with aqueous systems. Reviews on the aluminum battery are provided, e.g., with Li and Bjerrum (2002), Muldoon et al. (2014), Elia et al. (2016), Zafar et al. (2017), Zhang et al. (2018), and Nestler et al. (2019a). The search term “aluminum ion battery” leads to around 2,200 publications until 2017^16^, which is attributed to the imprecise definition of aluminum batteries and their mention in the context of high-valent batteries.

The first time that aluminum was reported as “une nouvelle application” for batteries (as positive electrode) in a galvanic pile together with zinc (mercury) in dilute sulfuric acid as the electrolyte—a primary cell—was communicated by M. Hulot (Dumas, 1855). As a negative electrode, aluminum was first utilized in the Buff cell in 1857 (Li and Bjerrum, 2002; Muldoon et al., 2014). In 1893, an amalgamated aluminum-zinc alloy was proposed for use as a negative electrode in a cell with carbon as a positive electrode (Li and Bjerrum, 2002), followed by the use of aluminum or amalgamated aluminum in heavy-duty chlorine-depolarized batteries (Li and Bjerrum, 2002). In the 1950s, aqueous electrolytes were considered for use in Leclanché battery cells with an aluminum negative electrode and a MnO_2_ positive electrode (Muldoon et al., 2014). While these different cells can be classified as primary cells, considerable research has been carried out to develop secondary aluminum batteries since the 1970s (Li and Bjerrum, 2002). Over the past 30 years, research efforts concerning rechargeable variants have encountered numerous problems, such as electrode material disintegration, low cell discharge voltage, capacitive behavior without discharge voltage plateaus, high self-discharge rate, insufficient cycle life with rapid capacity decay, and lack of rechargeability (Muldoon et al., 2014; Lin et al., 2015; Liu et al., 2017) as well as inconsistent research results (see below and Nestler et al., 2019a). In order to create an aluminum battery with a substantially higher energy density than a lithium-ion battery, the full reversible transfer of three electrons between Al^3+^ and a single positive electrode metal center (as in an aluminum-ion battery) as well as a high operating voltage and long cycling life is required (Muldoon et al., 2014). This has however, not been reported to date.

#### Aqueous or Primary Aluminum Battery

Despite its low cost, simple operation, and reduced environmental impact, aluminum batteries based on aqueous or protic systems exhibit fatal drawbacks, such as the passivating oxide film formation decreasing the battery voltage and efficiency, hydrogen side reactions, and material corrosion. The comparably low standard electrode potential of aluminum (−1.662 V vs. SHE) causes intrinsic hydrogen generation before aluminum could be plated in the process of reduction. This fact is indicated by the Pourbaix diagram (cf. Figure 3) and ultimately hinders the large-scale application of such systems (Zhang et al., 2018).

The protective layer can be removed chemically by changing the *pH* value of the electrolyte from neutral, by adding potent corrosive agents such as concentrated alkaline or acidic solutions. The electrode potential then restores to its thermodynamically allowed value. Additionally, this leads to an accelerated rate of wasteful corrosion (cf. Figure 3) and limits the battery shelf life (Muldoon et al., 2014). Thus, corrosion inhibitors have to be added in order to prevent loss of the electrolyte (Liu et al., 2017). The negative reduction potential of non-passivated aluminum-metal causes constant (parasitic) hydrogen evolution [see line (a) in Figure 3] when exposed to an aqueous/protic electrolyte solution.

A secondary aluminum-ion battery based on pure aluminum-metal as negative electrode and an aqueous electrolyte is unfeasible (Liu et al., 2017), because aluminum deposition only occurs at potentials far outside the stability region of water (see Figure 3). The electrolyte would decompose, and the ion transport gets disrupted. Primary (aqueous) aluminum batteries are summarized in Li and Bjerrum (2002). Theoretical specific energies of up to 1,090 Wh/kg are calculated, whereas real systems are reported to reach values of up to 200 Wh/kg. Both values are far below the theoretical specific energy of pure aluminum (Table 1). Such batteries are applied in the marine sector utilizing a complex (active) electrolyte supply and mixing system (Shuster, 1990; Licht and Peramunage, 1993; Li and Bjerrum, 2002).

Due to the inherent hydrogen generation of the aluminum electrode in aqueous electrolytes, a different battery design is needed, in which no metallic aluminum is used. Holland et al. (2018) proposes a design in which TiO_2_ is used as the negative electrode, CuHCF (copper-hexacyanoferrate) as the positive electrode, and an aqueous electrolyte consisting of AlCl_3_ and KCl dissolved in water. The authors concluded that Al^3+^ is the mobile species. The discharge voltage was reported to be 1.5 V, whereas the specific energy is 15 Wh/kg at a specific power of 300 W/kg with energy efficiency remaining above 70% for over 1,750 cycles. Since such a cell design utilizes a negative electrode comprising of other materials than aluminum-metal, the overall reachable energy density is limited.

#### Aluminum-Air Battery

The aluminum-air battery is composed of an aluminum-metal negative electrode, a positive electrode enabling oxygen transport and reduction, and a suitable electrolyte, typically alkaline solutions consisting of sodium hydroxide (NaOH), potassium hydroxide (KOH), or sodium chloride (NaCl) (Liu et al., 2017). Rechargeable variants were also reported utilizing non-aqueous electrolytes, such as ionic liquids (Liu et al., 2017). Up to now, these batteries are facing a series of problems such as the corrosion of aluminum, a high self-discharge rate, sluggish discharge kinetics, a principle lack of rechargeability, and short shelf life. If the “passivation” regime is chosen (cf. Figure 3), the oxide layer covering the surface of the aluminum electrode also decreases the cell voltage and cell efficiency in such batteries (Tang et al., 2004).

However, in 2014, Phinergy demonstrated that an EV equipped with a combination of an aluminum-air battery with a specific energy of (250–400) Wh/kg (at system level) (Yadgar, 2015) and a lithium-ion battery, which is charged by the preceding one, can cover a range of more than 3,000 km (Muldoon et al., 2014). Recently, an all-solid-state fiber-shaped aluminum-air battery with an electrode composed of cross-stacked aligned carbon-nanotube/silver-nanoparticle sheets with a specific energy of 1,168 Wh/kg was described (Xu et al., 2016).

#### Secondary Aluminum Battery

Secondary aluminum batteries are usually designed with an aluminum-metal negative electrode and a non-aqueous chloroaluminate-based ionic liquid electrolyte. These electrolytes have attracted increasing attention since 1988, when AlCl_3_ and imidazolium chloride were used due to their low vapor pressure and comparably wide electrochemical windows, enabling highly reversible stripping and plating efficiencies of aluminum (Zhang et al., 2018). Subsequent research applied the same electrolyte for secondary aluminum batteries because of the high success in lithium-ion batteries (Zhang et al., 2018).

In order to further develop rechargeable aluminum-ion batteries to make use of the full potential of aluminum, it is essential to develop electrolytes based on aprotic solvents stable against reduction by aluminum, enabling both aluminum deposition and dissolution (Muldoon et al., 2014). The other two stringent requirements for the electrolyte are a non-corrosive nature and a high resistance against oxidation. As indicated by line (b) in Figure 3 (here for water), oxygen can evolve from the electrolyte at the positive electrode during discharge. Therefore, an adequate positive electrode needs to be identified, with a standard potential inside the stability region of the electrolyte.

There are several options for potential novel electrolytes (Muldoon et al., 2014; Nestler et al., 2014; Elia et al., 2016; Liu et al., 2017). While liquid electrolytes pose the corrosion problems described above, solid electrolytes may solve these difficulties. However, due to the well-established aluminum electroplating industry, rich knowledge about electrolytes exists, offering impressive Coulombic efficiencies close to 100%. These electrolytes are often corrosive to battery components, which hinders discovery of high-voltage positive electrodes (Muldoon et al., 2014). The advantages of molten salts as electrolytes are high electrical conductivity, fast electrode kinetics and therefore less polarization, and high decomposition potential (Li and Bjerrum, 2002; Elia et al., 2016). Since aluminum can be electrodeposited from non-aqueous liquids, they are suitable for developing rechargeable aluminum batteries.

The electrolyte also determines, which species can be intercalated into the positive electrode. In Lin et al. (2015), a breakthrough was reached by assembling an aluminum battery with high-rate capability that uses aluminum-metal, a three-dimensional graphitic-foam as positive electrode, and a non-flammable ionic liquid as electrolyte. Instead of intercalating Al^3+^ ions, the ${\text{AlCl}}_{4}^{-}$ complex present in the ionic liquid was intercalated into the positive electrode (Nestler et al., 2019a). Such a cell would not make full use of the advantages connected to aluminum and should not be called an “aluminum-ion battery” but an “aluminum-chloride (-graphite) battery” (Kravchyk et al., 2017). Because of this monovalent chemistry, only positive electrodes with comparably low capacities (<100 mAh/g) have been found (Zhang et al., 2018). Computational research suggests that these complexes diffuse rather in tetrahedral than in planar form between graphite layers (Nestler et al., 2019a). This form of a hybrid/co-intercalation mechanism is, moreover, detrimental to the energy density. The cell exhibits well-defined discharge voltage plateaus near 2 V, a specific capacity of about 70 mAh/g and a Coulombic efficiency of approximately 98%. It can be cycled more than 7,500 times without capacity decay, has a specific energy of 40 Wh/kg (comparable to lead-acid and nickel-metal-hydride batteries, with a potential for optimization of the graphitic electrodes and development of other novel positive electrode materials) and a high specific power of up to 3,000 W/kg (similar to supercapacitors). A recently reported version (Zafar et al., 2018) uses a similar design but a commercial mesoporous carbon (CMK-3) as positive electrode. Such a battery shows a very long cycle life of >36,000 charge/discharge cycles with a high Coulombic efficiency of >97%, excellent charge/discharge performance of 50 C (3,000 mA/g), a specific energy of ~45 Wh/kg, and an average mid-voltage of 1.4 V.

Wang et al. (2016) reported another type of a rechargeable aluminum battery. It comprises a high-purity aluminum foil as negative electrode, a Ni_3_S_2_/graphene-microflakes composite-supposedly intercalating Al^3+^-as positive electrode, and AlCl_3_ dissolved in an ionic liquid of 1-ethyl-3-methylimidazolium chloride ([EMIm]Cl) as electrolyte. An initial discharge specific capacity of 350 mAh/g at a specific current of 100 mA/g is achieved. After 100 cycles, the discharge capacity remains over 60 mAh/g with a Coulombic efficiency of 99%. Later, they utilized a 3D-hierarchical copper sulfide (CuS) micro-sphere composed of nanoflakes as positive electrode (Wang et al., 2017). Such a battery then has an average voltage of ~1.0 V and delivers a specific capacity of about 90 mAh/g with nearly 100% Coulombic efficiency after 100 cycles at a specific current of 20 mA/g. In Jayaprakash et al. (2011), a similar design with a V_2_O_5_ nano-wire as a positive electrode was reported, delivering a discharge capacity of 305 mAh/g in the first cycle and 273 mAh/g after 20 cycles, with a stable electrochemical behavior, and an open circuit voltage of 1.8 V. The theoretical specific energy was determined to be 240 Wh/kg. Recently, the introduction of alternative urea-based chloroaluminate electrolytes as a way to circumvent corrosive ionic liquids as electrolytes was reported (Das, 2018). Here, a graphene positive electrode was used and characterized by the storage of trivalent aluminum ions at a relatively high operating potential. The aluminum-graphene cell offers the possibility of a high specific power (about 175 kW/kg), which is similar to that of supercapacitors, while the specific energy (about 66 Wh/kg) is higher than that of the lead acid battery. An extraordinarily fast recharge in the range of (1.1–60) s has been achieved with a specific capacity in the range of (60–110) mAh/g (Zhang et al., 2018).

In principle, there are two reversible mechanisms for the positive electrode: intercalation and conversion reactions. The former has been used in examples before, the latter is used in combination with positive electrodes based on sulfur. These offer the advantage of the transfer of two electrons during the electrochemical process, which allows for both lightweight and multielectron transfer materials simultaneously. Similar to lithium-sulfur batteries, non-aqueous aluminum pendants undergo a transition process of elemental sulfur to different polysulfide-chains due to electrochemical reduction of sulfur, eventually followed by conversion into Al_2_S_3_ (Zhang et al., 2018). Cohn et al. (2015) used sulfur as a positive electrode, but here, ${\text{AlCl}}_{4}^{-}$ ions have been identified as the mobile ionic species. This battery exhibits a discharge voltage plateau of ca. 1.2 V, with a very high charge storage capacity of more than 1,700 mAh/g, relative to the electrode of sulfur in the positive electrode. The specific energy of the Al/S cell is estimated to be 1,400 Wh/kg (sulfur). A low cycle efficiency of 4 was reported, due to the dissolution of sulfur containing intermediate discharge species into the ionic liquid electrolyte, resulting in the so-called “shuttle effect.” This rapid capacity degradation characterizes this type of battery as more of primary then of secondary type. Comparing intercalation-type with conversion-type electrodes, significant breakthroughs are possible through the complete utilization of a high-oxidation-state transition metal compound accompanied with a conversion mechanism. Thus, the identification of more conversion-type materials is of high importance.

### Materials

As summarized by Muldoon et al. (2014), for high-valent mobile species, the non-aqueous solid-state chemistry and electrochemistry is substantially more complex than for monovalent alkali metal species. Therefore, identifying and implementing a practical battery chemistry based on high-valent mobile species such as Al^3+^ is very difficult. The major developments needed for high-energy-density high-valent-metal batteries are rooted in the material discovery of both high-voltage positive electrodes and (solid) electrolytes. Significant issues include the compatibility of the electrolyte with the electrodes and the discovery of high-voltage positive electrodes capable of undergoing multiple electron transfers to the same metal center with rapid diffusion of the high-valent ion in the solid state. In the following section, characteristics and examples for the three main components of the aluminum-ion battery—the negative electrode, the electrolyte, and the positive electrode—are briefly discussed, whereas solid electrolytes are highlighted.

#### The Negative Electrode and Current Collectors

In order to make use of the full potential of the aluminum-ion battery, the negative electrode needs to consist of pure aluminum. The protective oxide layer on the aluminum surface, however, would be detrimental to the battery performance. It contributes to the fact that the reversible electrode potential is not achieved, and that the activation of the electrode is delayed (a time lag before the cell reaches its maximum operating voltage) (Li and Bjerrum, 2002). Any increase in the electrode potential is accompanied by accelerated wasteful corrosion in liquid electrolytes—aluminum undergoes a parasitic corrosion reaction, resulting in both <100% utilization of the electrode material and hydrogen evolution—and poor shelf life. This holds for aluminum-metal batteries with liquid electrolytes. Additions to the liquid electrolyte as well as the deposition of other oxide layers were done in order to significantly decrease the parasitic corrosion of the aluminum-metal electrode. Some groups report on benefits of the oxide film, such as the restriction of the growth of crystalline aluminum dendrites and strong surface corrosion, thus improving the cycling stability of an aluminum battery (Chen et al., 2017; Yoo et al., 2017).

For aluminum-air batteries the modification of the behavior of the oxide layer by means of specially designed aluminum alloys has been extensively explored (Li and Bjerrum, 2002). Aluminum alloys based on high-purity grade metals doped with elements such as Ga, In, Sn, Zn, Mg, Ca, Pb, Hg, Mn, and Tl have been investigated. Beside corrosion processes of pure aluminum, the electrochemical behavior of a number of aluminum alloys showing that alloying with certain metals can improve the voltage. For aluminum-ion batteries with aqueous electrolytes, it was found that the addition of a small amount of Zn, Cd, Mg, or Ba to the negative electrode lead to an increase in the electrode potential by (0.1–0.3) V, while the addition of Ga, Hg, Sn, or In gave an increase in the electrode potential by (0.3–0.9) V. Fan et al. (2015) investigated the electrochemical properties and battery performance of polycrystalline Al, Al (001), Al (110), and Al (111) single crystals. The study revealed that Al (001) single crystals displayed lower corrosion rate and higher capacity density due to the low surface energy.

Molten salts or other non-aqueous media like ionic liquids, provide an alternative electrolyte, in which aluminum does not form the surface oxide film and can be successfully electrodeposited from the electrolyte (Li and Bjerrum, 2002). However, the major obstacle in using ionic liquids is the lack of oxidatively stable, inexpensive current collectors that can operate in chloroaluminate ionic liquids. Wang et al. (2018b) present the use of titanium nitride as a compelling material for this purpose. Flexible current collectors can be fabricated by coating TiN on stainless steel or flexible polyimide substrates by low-cost, rapid, scalable methods such as magnetron sputtering. When these current collectors are used in a non-aqueous aluminum-chloride-graphite battery, stable cathodic operation is observed at voltages of up to 2.5 V (vs. Al^3+^/Al). Furthermore, those batteries have a high Coulombic efficiency of 99.5%, a specific power of 4,500 W/kg, and a cyclability of at least 500 cycles.

If solid electrolytes become available, the interface of the aluminum electrode on top of such a material is of great importance. The oxide surface layer of an aluminum electrode foil probably needs to be removed before it is joined with the solid electrolyte. Alternatively, a physical vapor deposition method could be utilized to deposit aluminum or, as discussed in Schnell et al. (2018), an infiltration of the solid electrolyte by liquefied aluminum.

#### The Electrolyte

A suitable liquid electrolyte in aluminum-ion batteries must serve both as a corrodent to dissolve the Al_2_O_3_ passivated layer and as a corrosion inhibitor for the other battery components. Aqueous systems suffer from poor cyclability, decomposition of the electrolyte, and hydrogen evolution, which can only be overcome by using a non-pure-metal electrode. Non-aqueous electrolytes, such as inorganic/organic salts dissolved in organic solvents, are highly flammable, and the efficient plating needs elevated temperatures (about 130°C) (Zhao et al., 2018). The ionic liquid (molten salt) electrolytes predominantly used at this moment are synthesized by mixing AlCl_3_ with organic salts, such as 1-butyl-3-methylimidazolium chloride (BMIC), 1-ethyl-3-methylimidazolium chloride (EMIC), or others (inorganic salts such as urea or NaCl) at a certain ratio. These can plate aluminum already at room temperature, prevent electrode corrosion, hydrogen evolution, and electrolyte drying. They do cause problems due to their air and moisture sensitivity and corrosion of other battery components (current collectors and housings) (Elia et al., 2016; Zhao et al., 2018). A combination of AlCl_3_ and EMIC is the most commonly used electrolyte in the literature, representing 67% of the studies. The other combination, AlCl_3_ and BMIC, is used in 10% of the cases. In addition, new and less expensive molten salt systems, such as AlCl_3_ combined with urea or systems supplemented with NaCl, have also been tested and represent 8% of the reported aluminum battery systems (Zhang et al., 2018). It has to be noted that polymer binders, such as polyvinylidene difluoride (PVDF), have been proven to be incompatible with chloroaluminate-based ionic liquids (Zhang et al., 2018). Moreover, the electrochemical window of these limits the output voltage of the positive electrode (≤2.4 V). Since typical AlCl_3_-containing imidazole-based ionic liquids are expensive, corrosive, sensitive, and with a low electrochemical window, they still remain far from commercial application (Zhang et al., 2018).

Inorganic solid electrolytes, in contrast, generally do not suffer from all these problems (Nestler et al., 2014) and exhibit advantages for safety and low cost. Moreover, they promise to increase both cycle life, due to their stability and energy density, because of generally higher electrochemical windows. Both aspects allow a more flexible choice of electrodes with higher voltages or capacities. Removing the need of the time-consuming electrolyte-filling step during battery assembly is another advantage from a technical point of view. Additionally, simplified battery structures can be designed: In the case of cell stacks, all batteries can be mounted in one container, instead of connecting individual containers as is necessary for the use with liquid electrolytes. More detailed advantages and also drawbacks can be found in Nestler et al. (2018).

Recently, a potential aluminum-conductive hybrid polyethylene (PEO)-oxide solid electrolyte was reported. Nanometer-sized SiO_2_ and the ionic liquid 1-ethyl-3-methyl-imidazolium bis(fluorosulfonyl)imide ([EMI]FSI) were used to plasticize the PEO and to improve the ionic conductivities of this material (Zhang et al., 2018). An ionic conductivity of 0.96 mS/cm can be achieved at room temperature, and an electrochemical window of 3 V (vs. Al^3+^/Al) is observed (Song et al., 2017). However, aluminum deposition and dissolution has not been found with such PEO-based electrolyte systems, possibly because the coordination of the ether group in the PEO with the ${\text{Al}}_{2}{\text{Cl}}_{7}^{-}$ species reduces the electrochemical activity (Zhang et al., 2018).

**Figure 6:**
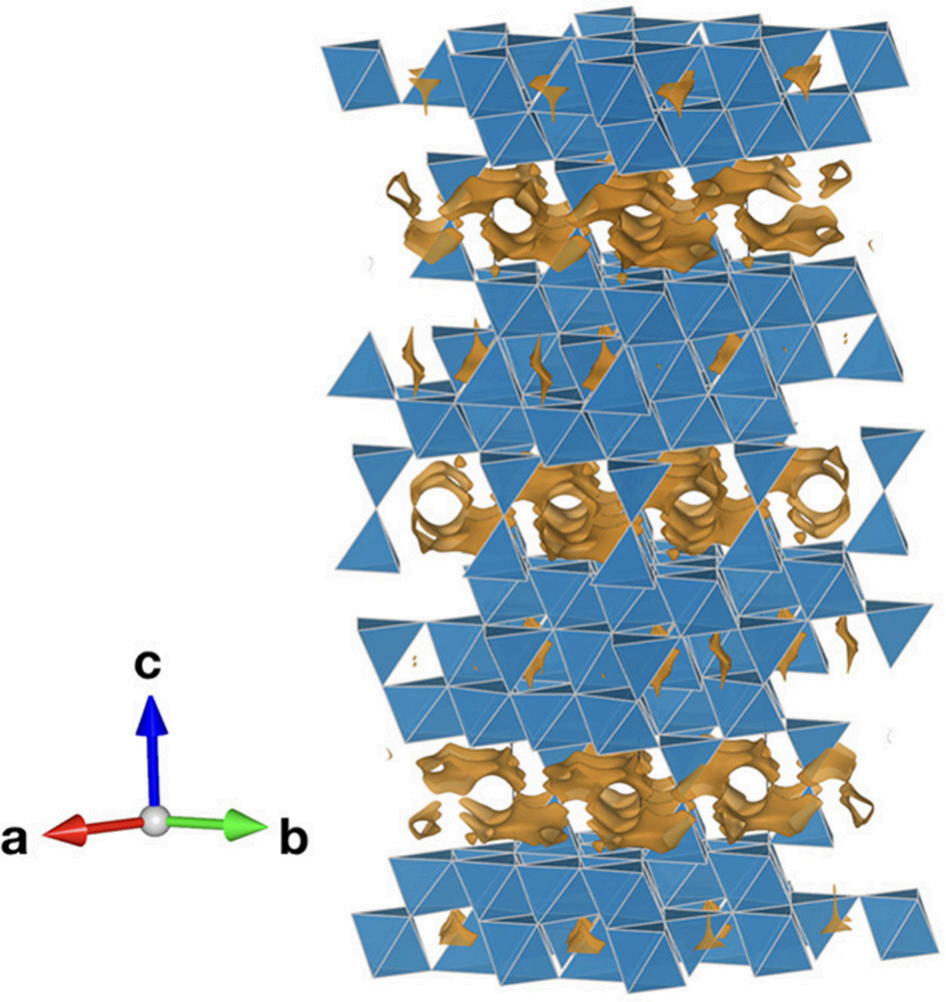
Bond valence energy landscape (orange) of Al^3+^ in Na^+^-β”-alumina with Na being removed and the Al kept for the calculation (Al_10.35_Mg_0.65_Na_1.65_O_17_, ICSD #6326592) drawn at the energy threshold of 1.07 eV. Blue polyhedra denote AlO_6_/AlO_4_/MgO_4_ octa- or tetrahedra. The calculations were performed according to Nestler et al. (2019b) using the program softBV (Adams and Rao, 2011).

For singly and doubly charged ions like Li^+^, Na^+^, K^+^, Ag^+^, Cu^+^, Mg^2+^, and O^2−^, different solid electrolytes are known. For Al^3+^, their existence is controversial (Nestler et al., 2019a). Different research groups claimed to have synthesized compounds that are able to conduct trivalent or even tetravalent ions. However, for instance *X*_2_(*B*O_4_)_3_-type compounds (*X* = Sc, Al, In, Lu, Yb, Tm, Er and *B* = Mo, W) have been proven by both theoretical and experimental work to show anion (dominantly O^2−^) instead of *X*^3+^ conduction. On the other hand, there are strong indications that β”-alumina with the general formula (Al_11−*y*_Mg_*y*_O_16_)(Na_1+*x*+*y*_O_(1+*x*)/2_) is indeed capable to conduct specific trivalent ions such as Gd^3+^, as was claimed by the group of Farrington in the 1980s (Nestler et al., 2019a; Figure 6). NaSICON (Na Super Ionic CONductor) materials, which can be described by the general formula *A*_*x*_*M*_*y*_(PO_4_)_3_, with *A* denoting an alkali metal ion (Na, Li) and *M* transition metals, are characterized by high ionic mobilities for the *A*^+^ ion. Substituting Zr^4+^ with smaller pentavalent Nb^5+^ can shrink the lattice (Nestler et al., 2019a). Furthermore, the higher valence state of Nb^5+^ in comparison to Zr^4+^ is thought to lead to effective reduction of electrostatic interaction between Al^3+^ cations and O^2−^ anions. A group claimed to have synthesized single phase NaSICON-type (Al_*x*_Zr_1−*x*_)_4/(4*x*)_Nb(PO_4_)_3_ for *x* ≤ 0.2, with the highest conductivity for (Al_0.2_Zr_0.8_)_20/19_Nb(PO_4_)_3_ with 0.45 mS/cm at 600°C (Nestler et al., 2019a). Trivalent ion conduction was investigated. However, a second group did not verify these results yet. Recently, the material (Al_0.2_Zr_0.8_)_4/3.8_NbP_3_O_12−x_F_2x_ (0 ≤ *x* ≤ 0.4) was synthesized, whereas the F^−^ doping was meant to increase the ion conductivity to 1.53 mS/cm at 500 °C, with ion transference number higher than 0.99 at (300–700) °C (Wang et al., 2018a).

Additionally, the fact that Al^3+^ ions have been proven to intercalate in the chevrel phase Mo_6_S_8_ (see below), suggests that finding ionic conductors with considerable aluminum mobility is plausible, despite the expected strong Coulomb interactions with the host lattice that should induce higher migration barriers than for di- or monovalent ions in the same lattice framework and the low polarizability of Al^3+^ (Nestler et al., 2019a). Thus, it is more difficult to find suitable host structures in comparison to monovalent ions. This assumption is underlined by comparing the bond dissociation energies (Haynes, 2011). Due to its low electronegativity and high charge of the trivalent ion, aluminum shows rather high dissociation energies to higher-electronegative non-metals (and thus possible counter-anions). These energies are especially pronounced for the halogens (502–675 kJ/mol) and oxygen (502 kJ/mol). The lowest bonding energies can be found for the group V elements like phosphorous (217 kJ/mol) and arsenic (203 kJ/mol). In the best scenario, probably, third elements (other cations in the structure) should show higher bonding energies to the counter-anions than aluminum and the bonding energy of aluminum to the counter-anions should be low even on an absolute scale. The less aluminum interacts with its surroundings, the better. On the other hand, calcium, magnesium, and lithium for which several oxide intercalation materials and solid electrolytes are already known, display higher dissociation energies (383–341 kJ/mol), which also encourages exploration in this direction.

Chemical and crystallographic aspects for fast ion transport have been comprehensively discussed and summarized with a focus on high-valent ions, including Al^3+^ (Nestler et al., 2019a). Especially the screening of the Coulomb interactions by ions in the lattice with a higher same-sign charge as the considered mobile ion should be an important prerequisite. For aluminum, this can be achieved e.g., by incorporating W^6+^, Mo^6+^, Mn^4+^, P^5+^, or Si^4+^ in the respective materials. Furthermore, the presence of other ions with a lower net charge in the compound has to be examined critically due to their higher likelihood of migration. Thus, it seems that aluminum conductors have to be at least ternary compounds containing a transition metal or other high-valent positive ions. For crystalline materials, the ones with high symmetry should be preferred, since it induces 3D pathways as well as channels that are more likely to contain sites with similar anion coordination and site energy (Meutzner et al., 2015). In general, for fast ionic transport, sulfur compounds would be preferable, since sulfur is more polarizable and is larger than oxygen and would thus decrease the electrostatic interaction with aluminum. Even though sulfides seem to be promising as positive electrodes, sulfide-based solid electrolytes in general possess a significantly smaller electrochemical window and tend to be unstable in air, sensitive to moisture, and likely to react with adjacent materials. Since there is still a lack of solid electrolytes for Al^3+^ conduction, below an approach for finding such materials is presented as well as the results thereof.

In order to compete with lithium all-solid-state batteries, ionic conductivities above 0.1 mS/cm over a large temperature range are demanded (Schnell et al., 2018). The question whether comparatively high mobilities for Al^3+^ in solids appear plausible at all can thus be approached best through analyzing intercalation electrode materials at the moment (see below). Conversely, the discovery of novel electrode materials can certainly be accelerated by the identification of novel electrolytes.

#### The Positive Electrode

Beside the electrochemical processes, positive electrodes should fulfill specific properties in order to meet the techno-economic requirements. Here, oxide materials represent the upper boundary for the energy density (Canepa et al., 2016). Utilizing the BatPac-Model for an aluminum battery, the positive electrode should have a density larger than ca. 4 g/cm^3^, the open-circuit-voltage (OCV) should be around 2.5 V or the density must be larger, in order to meet, e.g., the United States Advanced Battery Consortium goals (Canepa et al., 2016).

**Figure 7:**
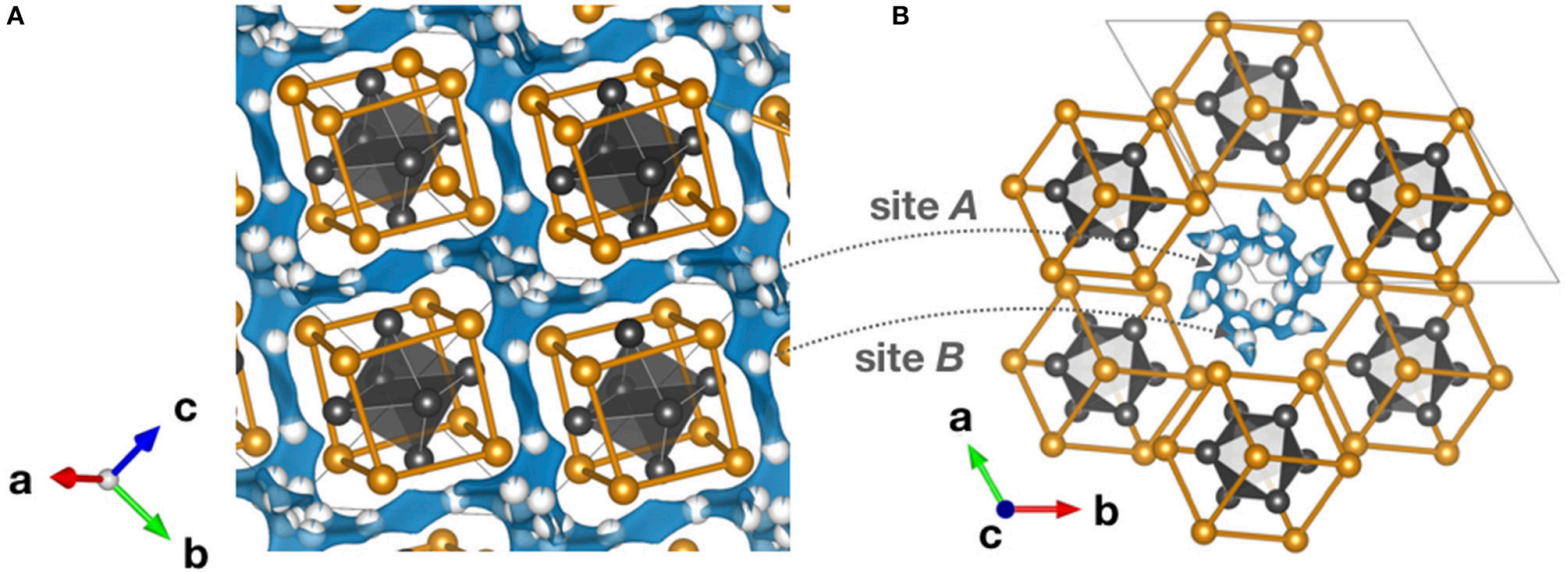
Bond valence energy landscape (blue) for Al^3+^ intercalated chevrel Mo_6_S_8_ (ICSD #158986 with cell parameters from Levi et al., 2007). They are drawn at energy thresholds of **(A)** 0.33 eV to show the whole path and **(B)** 0.23 eV to show the match of the sites suggested by the bond valence method and Al sites (partially blue) reported in literature. S anions forming cubic cages are marked orange, while the enclosed Mo_6_ octahedra and the unit cell are gray. In **(B)** the six atomic sites of the inner and the six of the outer rings are illustrated. The calculations were performed using the program softBV (Adams and Rao, 2011).

Studying the literature, reports of positive electrode materials for secondary aluminum batteries, which operate by reversible electrochemical intercalation of Al^3+^, have been scarce but are on the rise since the last couple of years (Muldoon et al., 2014; Nestler et al., 2019a). Indeed, all the suggested compounds are already known from lithium, sodium, or magnesium-ion battery research and it appears that only a few reveal an unambiguous reversible intercalation of Al^3+^. The strongest indications of Al^3+^ intercalation have been found for layered TiS_2_, chevrel-phase Mo_6_S_8_ (Figure 7), V_2_O_5_, graphite, Prussian blue analogs, and various manganese oxides (Nestler et al., 2019a). Various conversion materials such as metal halides (Hg_2_Cl_2_, AgBr, NiCl_2_, CuF_2_, and FeCl_3_) have also been reported to react with Al^3+^ (Nestler et al., 2019a). Some of them have also been demonstrated as positive electrodes for magnesium batteries. Unfortunately, aluminum batteries containing these electrodes are plagued by low OCV when compared to their magnesium counterparts (Muldoon et al., 2014). In the case of the aluminum counterpart, while the electrode's specific capacity was similar to that obtained with magnesium, the operating potential was below 0.5 V (Muldoon et al., 2014).

Conversion electrodes offer voltages slightly below 1 V with aluminum, but their solubility in liquid electrolytes may result in direct reaction with the aluminum electrode, which limits cell cyclability (Muldoon et al., 2014). Improvements in terms of voltage and capacity are ongoing; a capacity of 273 mAh/g for V_2_O_5_ at an average discharge voltage of 0.5 V with good cyclability (20 cycles) has been observed and the use of amorphous V_2_O_5_ with a similar discharge voltage vs. Al^3+^/Al was also reported (Muldoon et al., 2014).

Other electrode materials comprise TiO_2_ nanotube arrays (Liu et al., 2012) and a graphene electrode (Das, 2018). Here, graphene was characterized by the storage of Al^3+^ ions at a relatively high operating potential. It is superior to other such materials in terms of its high energy density, power density, lifetime, thermal stability, reliability, and flexibility.

In general, the rather ionic character of common transition-metal oxides enables a high-valent intercalation at desirable high voltages at the cost of reduced bulk diffusion properties (Canepa et al., 2016). The electrodes can be utilized in nanostructured forms that will help reduce the impact of slow solid-state diffusion as well as structural distortion during intercalation/deintercalation (Muldoon et al., 2014).

Up to now, the only studies that truly prove reversible Al^3+^ ion intercalation concern the chevrel phase Mo_6_S_8_ (Nestler et al., 2019a). In general, the chevrel structure can be envisioned as a stacking of Mo_6_S_8_ blocks that are composed of octahedral Mo_6_ clusters with strongly correlated 4*d* electrons. These clusters are embedded in S_8_ anion cubes. The arrangement of the building blocks forms a three-dimensional channel system of face-sharing pseudo-cubic cavities. It offers two different insertion sites for cations: six positions in the so-called inner ring (site *A*) and another six positions in the outer ring (site *B*) located above and below the inner ring. X-ray diffraction and galvanic intermittent titration studies suggest two insertion sites for Al^3+^. The chalcogenide-based chevrel-phase-electrode enabled the realization of the first working magnesium battery in 2000 (Aurbach et al., 2000).

Prussian blue analogs have been demonstrated to reversibly intercalate a multitude of high-valent ions including Al^3+^ from both aqueous and non-aqueous electrolytes (Canepa et al., 2016). Excellent electrochemical cyclability was demonstrated (2,000 cycles without capacity fading) for Al^3+^. However, the insertion voltages [(0.60–1.3) V vs. SHE] as well as the specific capacities of (30–60) mAh/g are poor, resulting in energy densities of 102 Wh/kg and 171 Wh/l that are far-off from the expectations of a practical high-valent battery (Canepa et al., 2016).

**Figure 8:**
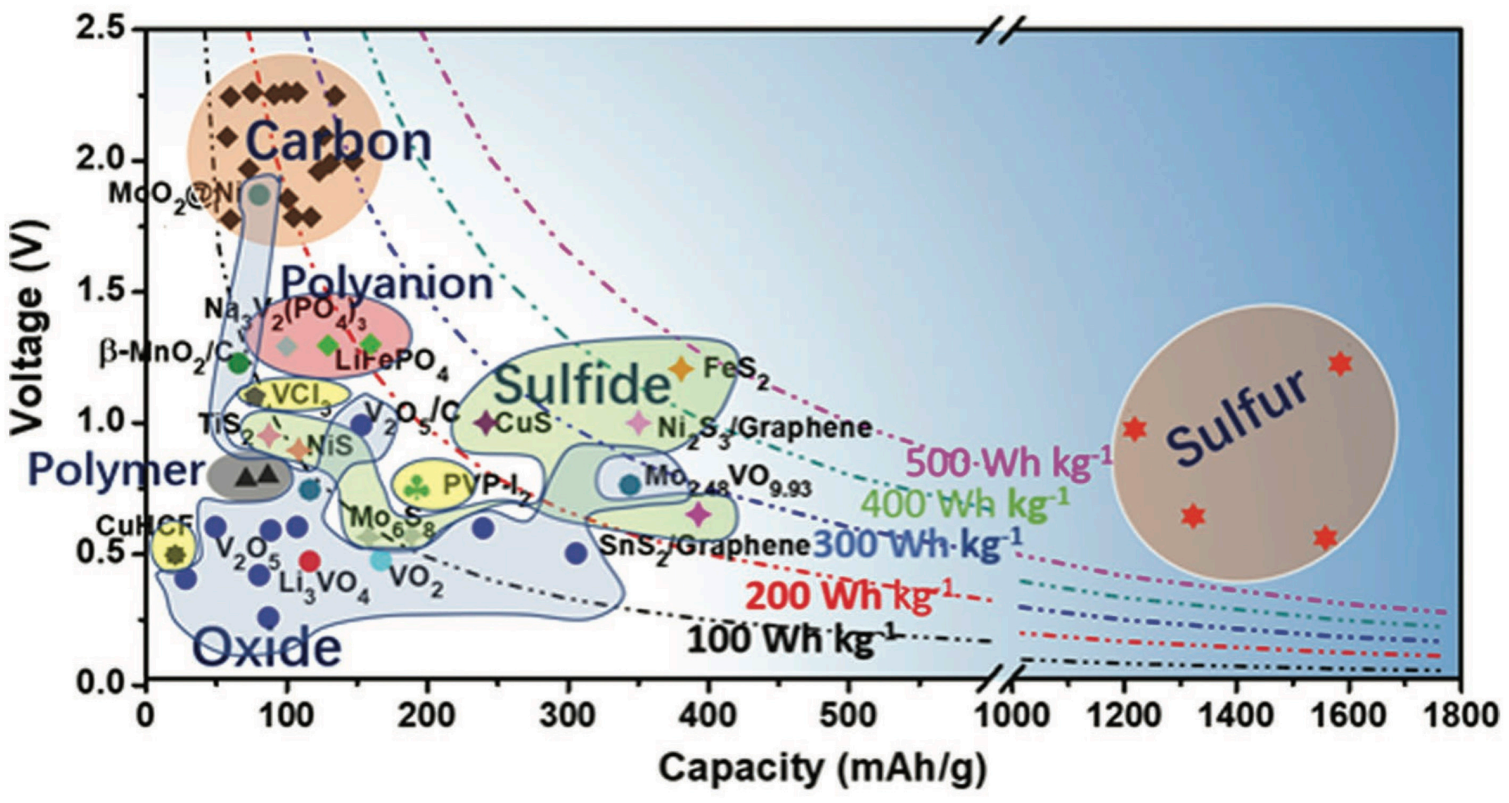
Capacity vs. voltage for reported positive electrodes for non-aqueous aluminum batteries. The dots present the practical average discharge voltages and the corresponding available specific capacities of each material. It has to be noted that these materials have not necessarily been proven to intercalate Al^3+^ but chloroaluminate ions, such as ${\text{AlCl}}_{4}^{-}$, instead. Figure reproduced with permission from Zhang et al. (2018) © 2018 WILEY-VCH Verlag GmbH & Co. KGaA, Weinheim.

A collection of so far utilized positive electrodes for aluminum intercalation (mostly chloroaluminate ions), the used electrolytes, discharge capacities, and discharge voltages as well as other parameters can be found in Nestler et al. (2019a), Xing et al. (2018), and Zafar et al. (2017). Figure 8 provides a summary of most reported positive electrodes. Accordingly, besides sulfur (Nestler et al., 2019a) and FeS_2_ (Mori et al., 2016)—showing an initial gravimetric capacity of 1,310 mAh/g and 610 mAh/g, respectively—three-dimensional carbon-encapsulated cobalt selenite nanoparticles developed from metal organic frame-works seem to be promising (Xing et al., 2018). This material showed a gravimetric capacity of 427 mAh/g at a specific current of 1,000 mA/g with two high discharge plateaus at 1.0 and 1.9 V (equivalent to an energy density of 424 Wh/kg) and a retained capacity of 16% after 100 cycles. However, all these materials have not been verified unambiguously for Al^3+^ intercalation so far. Co-intercalation as well as side reactions are often thought to be responsible for the observed capacities.

### Identifying Novel Materials

As stated by Canepa et al. (2016), the discovery of positive electrodes (and solid electrolytes) remains one of the main obstacles toward high energy-density high-valent battery prototypes. Oxides theoretically provide improved energy density at the expense of ion mobility. “Fortunately, the vast chemical space of possible positive electrodes for high-valent ions remains largely unexplored and predictions of high mobility in new material classes such as post-spinels, silicates, and fluoro-polyanions suggest that a covenant of peace between the two contending parties, kinetic performance and energy density, can be simultaneously attained” (Canepa et al., 2016).

**Figure 9:**
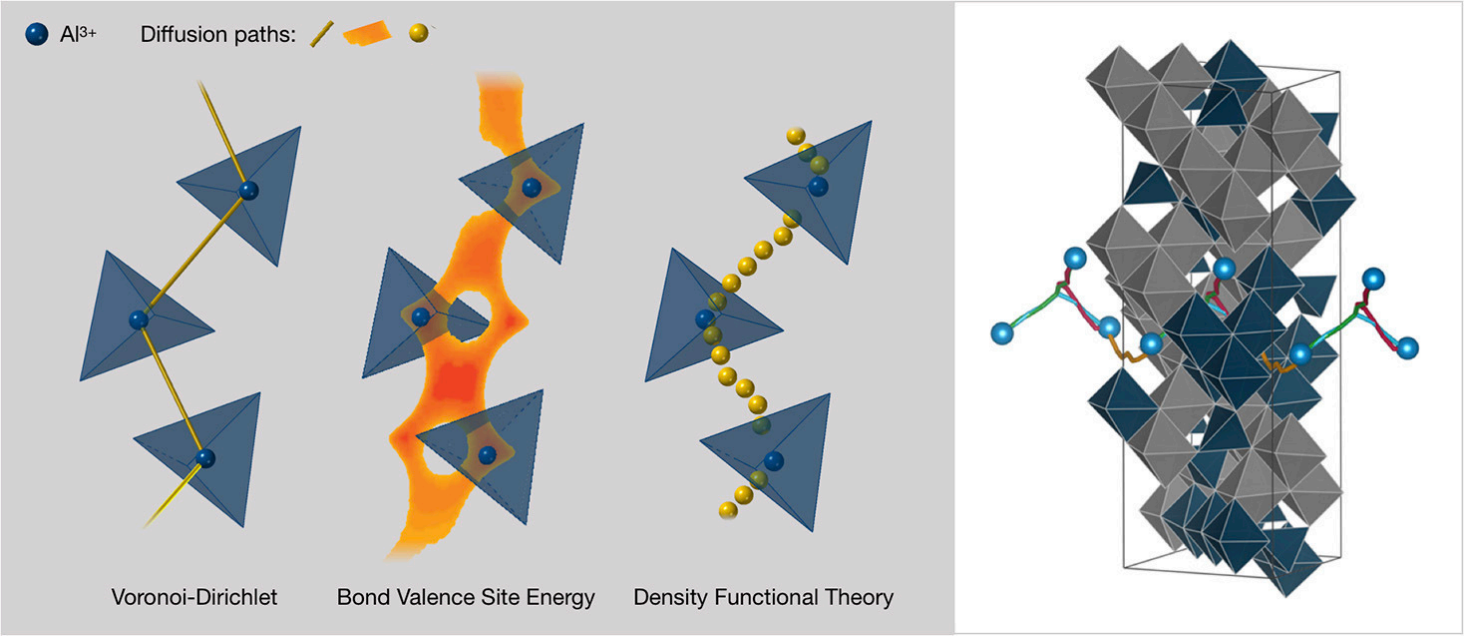
Scheme of the suggested approach for identifying crystalline materials with fast ionic conduction for aluminum-ion battery materials: Voronoi-Dirichlet partitioning, bond-valence site-energies, density-functional theory (NEB) analysis. Simulation methods with different accuracy levels and thus computational effort, are performed in succession. Right: Al^3+^ pathways investigated by DFT-NEB analysis in the 1 × 1 × 3 AlVO_3_ supercell (Nestler et al., 2019b). All pathways are connected via a nearest-neighbor, unoccupied aluminum site. Blue octahedra represent AlO_6_, while gray octahedra are VO_6_. Blue balls represent the start and end positions in the investigated paths.

**Figure 10:**
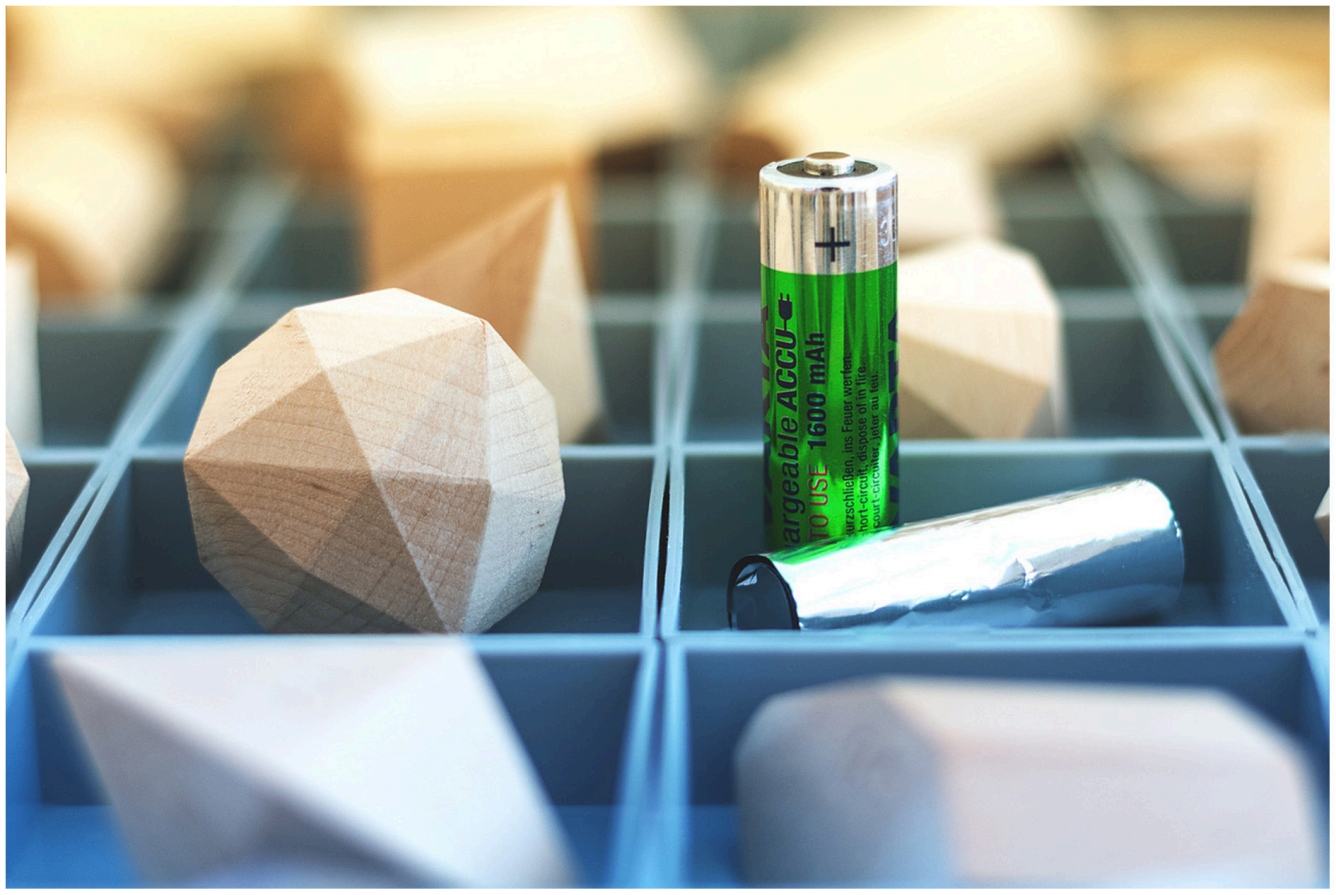
Using crystallography, crystallographic databases, and data mining with crystallochemical methods for the identification of novel aluminum ion conductors (positive electrodes and solid electrolytes).

Accordingly, it has not been possible to exploit the technological potential of the aluminum-ion battery, as suitable materials were lacking. The hope to be able to transfer compounds or at least structural motifs from the lithium-ion battery to higher energy density battery materials such as for magnesium (Levi et al., 2009) or aluminum-ion batteries (Elia et al., 2016) also often failed. Thus, the discovery of inorganic materials with high aluminum-ion mobility is a necessary innovation leap forward in the field of rechargeable aluminum- and high-valent-ion batteries. By analyzing the necessities for good ionic transport and applying this knowledge to large crystallographic databases by means of high-throughput crystal-chemical analyses applying an established combined approach of different theoretical methods (see Meutzner et al., 2015, 2017, 2018a; Meyer et al., 2018; Nestler et al., 2019b; Figure 9), we address the identification of promising materials' candidates for a future all-solid-state aluminum-ion battery technology (Figure 10).

#### Theoretical Approach

Diffusion is a process on the atomic scale; a specific ion moves from one place, in which it is coordinated by a specific set of atoms, to another place, in which it is surrounded by another specific set of atoms. In solid-state diffusion, these coordination spheres are fixed in space and usually show regularities, especially in crystalline compounds. This hopping mechanism involves thus more energetically stable sites (the starting and end points of the movement) and those in-between, usually less energetically stable sites (Zschornak et al., 2018). This rather simple process can be modeled by a variety of methodologies. Especially *ab initio* methods allow a calculation of the energies of the various (sub-) steps of diffusion through the solution of the Schrödinger equation for all electrons in the system. The widest-spread of these methods applies density functional theory (DFT) using the so-called nudged elastic band (NEB) algorithm (Hohenberg and Kohn, 1964; Kohn and Sham, 1965; Sheppard et al., 2008; Jain et al., 2013; Johannes et al., 2016). It tries to find the energetically most favorable reaction path between two sites. These calculations are computationally very expensive and should be carried out only for materials, in which conduction is deemed probable.

Using computationally less expensive methods, on the other hand, allows screening of material databases, which may have never been analyzed for these properties but show similarities to other materials with these features. Voronoi-Dirichlet partitioning (VDP) fractionizes any given (crystal) structure using a simple geometric construction: (1) create a line between a point (atom) and any other neighboring point (atom); (2) construct a face perpendicular to this line, exactly in the middle of these two points; (3) in doing so, a smallest possible polyhedron is generated for the initially chosen point, its Voronoi-Dirichlet polyhedron (Blatov, 2004). These polyhedra comprise the space closer to this (central) point than to any other point and the outer boundaries can be interpreted crystallochemically as possible void sites (vertices), connecting lines (edges), and strength of the interatomic connection (face) as well as the size of this atom in this structure (volume of the polyhedron). By analyzing the thus identified voids' sizes and connectivities, applying a prior data mining, ion conduction pathways can be uncovered in crystalline materials (Blatov et al., 2006). These calculations are very fast and can be easily applied to crystallographic databases (Anurova et al., 2008; Meutzner et al., 2017; Eremin et al., 2018). This way, a subset of interesting materials can be preselected and the results refined by other modeling routine like bond-valence site-energies (BVSE) before actually utilizing DFT-based calculations.

After an exponential relation between bond-length and bond-strength was observed in mostly ionic crystalline compounds, these ideas were primarily used to check the chemical plausibility of crystal structures. Additionally, sites occupied by the lightest elements, like hydrogen and lithium, which cannot be detected by X-rays, could be identified (Brown, 2009). This idea was later revisited in the framework of ionic conduction and extended by: (1) taking post-first-coordination-shell contributions into consideration (Adams, 2001); (2) using relative bond-valence sum (BVS) mismatch maps (Adams and Rao, 2009); (3) eventually translated into an energy via a Morse-type potential (Adams and Rao, 2011). Still based on the static crystal structure reported within the literature, these BVSE allow a more chemically reasonable consideration and even the calculation of migration energies but are computationally more expensive than VDP.

We thus recommend a successive application of increasingly complex calculations to identify potential ionic conductors (Figure 9). Firstly, a subset of compounds is chosen from the database comprising aimed-at materials, usually the mobile ion (e.g., a cation like Al^3+^) and the desired counter-ion (e.g., an anion like O^2−^ or I^−^). These compounds are then analyzed by VDP, followed by BVSE calculations. Eventually, the most-promising compounds are modeled by DFT-NEB in order to combine the time and computational cost advantages of each of the methodologies. This approach is described in detail in Meutzner et al. (2018b) and Nestler et al. (2019b).

#### Novel Materials

**Figure 11:**
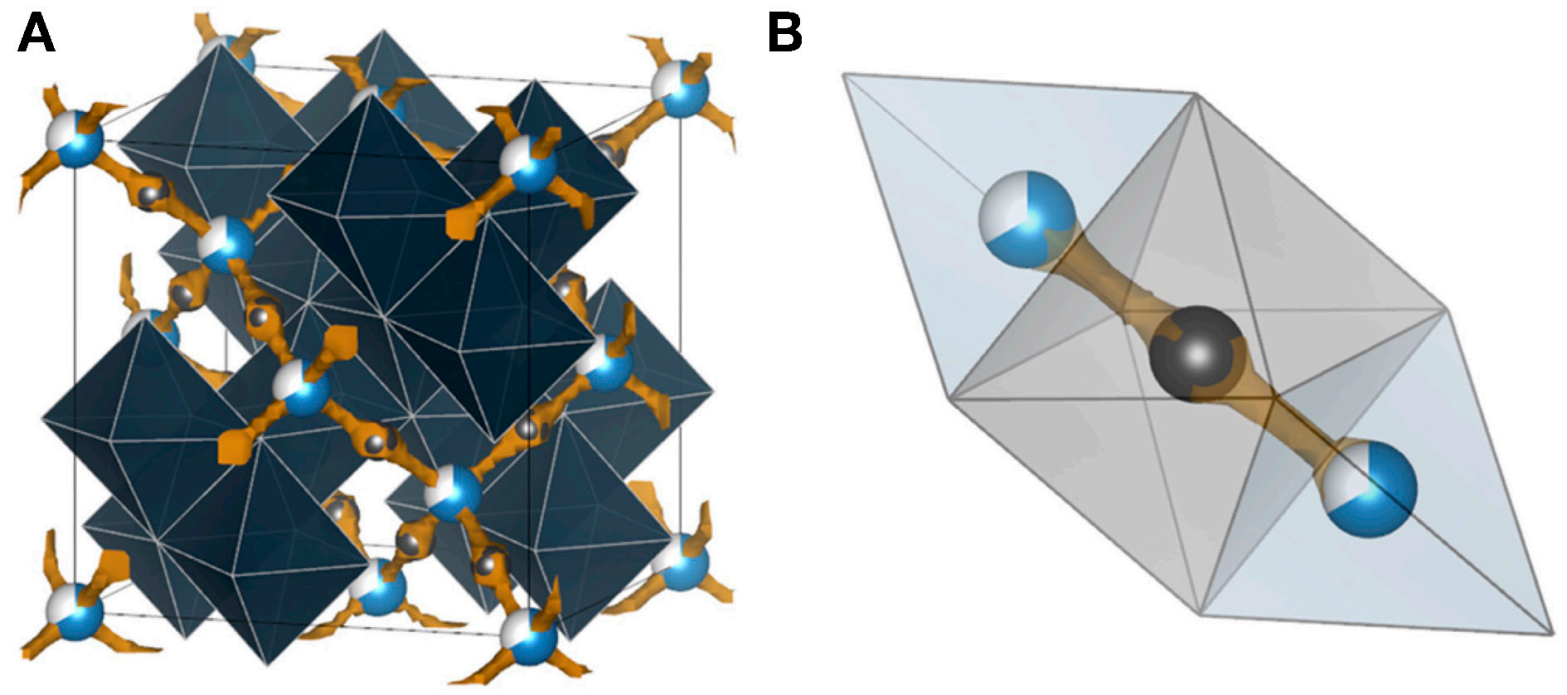
Bond valence energy landscape (orange) for Al (light blue) at 0.52 eV in AlVO_3_ (ICSD #49645). **(A)** Dark blue polyhedra denote the 16*d* site, which is occupied by V (2/3) and Al (1/3) and coordinated by eight oxygen atoms. Half of all Al in the structure occupies the 8*a* site and is suggested to be part of a 3D pathway. This network coincides with the hopping positions calculated by VDP (gray). **(B)** For aluminum migration, an octahedral site (gray) has to be passed during migration. Reprinted with permission from Nestler et al. (2019b). Copyright 2019 American Chemical Society.

Applying the described approach, several promising materials can be suggested (Meutzner et al., under review; Nestler et al., 2019b; Rothenberger, 2019), which are summarized in Table 3. Recently, the authors have proposed the oxide AlVO_3_ (Figure 11) as a promising novel candidate as positive electrode (Nestler et al., 2019b). So far, compounds comprising at least Al and O (as well as O-containing without Al), Al and S, and Al and Se have been investigated, while the whole ICSD is screened for further suitable compounds.

**Table 3:** Promising materials as positive electrodes or solid electrolytes for rechargeable aluminum-ion batteries.

**Compound**	**ICSD-#**	**Migration Barrier (eV)**
Al_2/3_(Al_2/3_V_4/3_)O_4_	49645	0.52 (3D)
Al_2_S_3_	25352	0.50 (3D)
Al_2_Se_3_	14373/25353	0.40 (3D)
Tl_3_Al_13_S_21_	71756	0.45 (3D)
AlPS_4_	428184	0.45 (2D)
Al_0.44_La_3_Si_0.93_S_7_	91326	0.56 (1D)
AlI_3_	391247	0.209 (3D)
AlBr_3_	39768	0.308 (3D)
Ni(AlCl_4_)_2_	417872	0.325 (2D)
Ti(AlBr_4_)_2_	40904	0.335 (2D)
V(AlCl_4_)_2_	415951	0.335 (2D)
AlP	609019	0.615 (3D)

*Given are the composition (Compound), the ICSD collection number (ICSD-#) (FIZ Karlsruhe GmbH, 2018), and the migration barriers and respective dimensionalities calculated by the BVSE method using the program softBV (Adams and Rao, 2011)*.

Due to the strong interaction of Al^3+^-ions with their atomic environment, the authors studied the bonding and possible shielding effects induced by higher-valent cations in the extended chemical neighborhood of aluminum. In fact, we found a decrease of charges and an increasing displacement of the chalcogenides toward higher-valent ions for the heavier chalcogens, which we interpret as a decrease of the electrostatic interaction (Meutzner et al., under review). In fact, in Canepa et al. (2016), it was already proposed that the increased covalency and larger volume per anion of chalcogenides, in comparison to oxides, tend to decrease the electrostatic interactions between the diffusing high-valent ion and the anion lattice, thus reducing the migration barriers. Accordingly, the ion polarizabilities, the ability to deform the anion electronic charge density by an external electric field or potential (e.g., the charge of a mobile ion, such as Al^3+^), increase moving down the chalcogenide group.

## Critical Analysis of the Current State of Knowledge

As indicated above, until 2017, just around 2,200 articles concerning “aluminum batteries” but only around 120 articles concerning the “aluminum-ion battery” have been published. At least six review articles, like Elia et al. (2016), Liu et al. (2017), Zafar et al. (2017), Zhao et al. (2018), Zhang et al. (2018) and Nestler et al. (2019a). were published in the last 3 years, alone. The amount of manuscripts reporting fundamentally new insights, materials, or even technological jumps was relatively low (e.g. Lin et al., 2015; Wang et al., 2017; Xing et al., 2018).

The amount of dedicated funding programs steadily increased to this day and energy storage and energy materials are usually a “hot topic” for these kinds of third-party funding calls. The lithium-ion battery technology achieved its big success only in the last decade. Therefore, no other nations beside Japan, later South Korea, and now China entered in the quickly growing battery market and could thus not establish or expand their own battery research and development. The aluminum-ion battery with its difficulties and low exploration thus was the least to be considered in funding and research so far. Globally, the battery field and especially high-valent chemistries received several strong impulses in the past decade, in particular by the increasing discussions concerning climate change and energy supply. Especially the nuclear plant accident in Japan in 2011 and, for instance, the intensification of the German *Energiewende* (energy transition), as well as efforts of Tesla Inc. raised the awareness for this topic.

In science, as it is conducted as of today, usually and unfortunately, only successful results are published. Mostly, the unsuccessful attempts are not reported. For this reason, it seems very probable that the same materials are tested by multiple working groups within the scientific community, potentially leading to highly redundant research and time that would have been better spent for continuing assessments or experiments of different new materials. The authors, for instance, chose Al_2_(WO_4_)_3_ as solid-electrolyte for an all-solid-state aluminum-ion battery. This material was elaborately synthesized and crystallographically as well as electrochemically analyzed in half- and full-cell configuration. Unfortunately, we could observe no Al^3+^ ion conductivity and thus no energy conversion. An intensified literature study revealed omitted or ignored references in many articles. Even though other work groups showed that this material was no Al^3+^ ion conductor, further manuscripts got published, still claiming the outstanding conducting properties (Nestler et al., 2019a). Even referees are not aware of the whole story and taking this controversial work into account when reviewing manuscripts. As another example, a couple of works have been published, reporting an intercalation of Al^3+^ or at least Al^3+^-containing compounds showing X-ray diffraction (XRD) studies without differences in pre- and post-intercalation states that are attributed to the small Al^3+^-ion or its low concentration to which XRD is very well capable of detecting the difference (Liu et al., 2012; Xing et al., 2018). An electrochemical reaction was observed, but the analysis seems incomplete. We think, there may rather be adsorption/chemisorption and a following accumulation on the surface of the microstructures involved, which would also be good for electrochemical energy storage, but a different optimization strategy would have to be applied. In the bulk of the corresponding publications, no explanation is given as to why the respective material was chosen, which materials have been screened beforehand, and what was the deciding point for the sub-amount of screened compounds.

Current research thus evokes the feeling of randomness of the investigational directions taken; why certain compounds were tested and others not. This approach depends more on chance than on an actual research strategy. To our opinion, therefore, systematic theoretical and experimental studies are necessary, and we want to encourage this course of action, rather, with this review article and the suggested algorithm.

Realizing an aluminum-ion battery will necessitate a high experimental and financial effort. Therefore, we propose an interdisciplinary collaboration for the physical and/or chemical synthesis of predicted materials [maybe also with special methods like ionic liquids (Ahmed and Ruck, 2011), or utilizing MOFs (Xing et al., 2018)] in bulk, powder, and by thin-film technology. The efforts and competences should be bundled and coordinated; for example, a society with industry partners could be founded at least on a national level. Furthermore, research should become more standardized and adequate methods should be mandatory for the verification of actual ion conductivity, like the Tubandt method, GIIT, and electrochemical impedance spectroscopy for solid electrolytes. In the end, it will be the decision of the scientist if he or she wants to pursue a resource-efficient concept of perhaps lower performance or live with the risk of utilizing scarce or risky raw materials.

## Summary and Outlook

Research on the aluminum-ion battery is currently experiencing a strong intensification worldwide, especially in China. However, most of the studies are dealing with aluminum-chloride-graphite batteries instead of aluminum-ion batteries, since ${\text{AlCl}}_{4}^{-}$ instead of Al^3+^ is the mobile species. Current market studies already consider the aluminum-ion battery technology as worth for investigating as an important post-lithium concept. This is not only based on significantly increasing the (theoretical) energy density compared to today's lithium-ion batteries, but also refers to the good availability of the raw material aluminum as the most abundant metal in the earth's crust, the already available aluminum value chain and infrastructure, including recycling, and the high safety of aluminum-ion all-solid-state concepts. Patenting related key-technologies and materials as a starting point for successful market placement is still possible because there are very few patents, currently.

At this moment, the advantages mentioned, and the high dynamics in the field of the aluminum-ion technology point to the growing interest in this field. In order to solve the challenges of developing a rechargeable battery and bringing it to the market, a swift, concerted procedure with specific know-how, competencies, and resources, and interdisciplinarity of science and industry is required, emphasizing the electrolyte and the positive electrode. On the one hand, the work is to be aligned along an innovation chain, from physics and chemistry to materials science and from materials science to technology. On the other hand, there is an already established closed value chain, from raw material preparation, material production, further processing, and component and system development, through applications up to recycling, which has to be incorporated into the innovation process. There is a patenting strategy and a roadmap to develop in order to define the main research activities, milestones, and lines and to fit these into a timely framework.

The main challenges to overcome are the lack of compatible (solid) electrolytes and poor Al^3+^ mobility in many electrolytes and positive electrodes. The resulting current aluminum batteries suffer from poor energy densities, necessitating the exploration of alternative materials in particular for setting up the aluminum-ion battery. Further challenges are connected to the oxide layer of the metal electrode and the interfaces between negative electrode, solid electrolyte, and positive electrode. The limits concerning interfaces and diffusivities, however, might be overcome with an entirely new manufacturing route making use of thin-film technologies already existing in the semiconductor industry. Hence, suitable working solid aluminum-ion conductors could push the development of rechargeable aluminum-ion batteries. At the same time, they would pave the way to build thin-film batteries with extraordinary energy densities, applicable for the on-chip power supply of sensors and mobile applications.

Most important seems to be the identification and prediction of new materials, a collection (data base) of investigated materials deemed both promising and not promising.

Coming back to the title of this article questioning “The aluminum-ion battery: A sustainable and seminal concept?” we can answer that, indeed, the aluminum-ion battery is a highly promising battery technology concept. If progress is achieved in reversible aluminum stripping and deposition as well as in identifying suitable (solid) electrolytes and positive electrodes, this battery may open up a huge application range.

## Author Contributions

TL conceived the presented idea and took the lead in writing the manuscript together with FM. All other coauthors contributed in their respective fields and added further ideas: FM with Voronoi-Dirichlet partitioning, MZ with solid-state physics, WM with lithium-ion battery research, RS with economic aspects of the battery market, TN with bond valence methodology, RE and AK with DFT modeling. VB and DM encouraged and supervised the findings of this work. DM supervised the project from which this article was motivated. All authors discussed the results and contributed to the final manuscript.

### Conflict of Interest Statement

The authors declare that the research was conducted in the absence of any commercial or financial relationships that could be construed as a potential conflict of interest.

## Footnotes

^1^www.sciencenewsforstudents.org/article/really-learn-fail-then-fail-again (December 2, 2018).

^2^India, the Netherlands, the UK, and France, for instance, want to exit combustion technology in 2030 or 2040, whereas, e.g., Volvo wants to sell only hybrid and full electric vehicles from 2019 on.

^3^https://www.stortinget.no/no/Saker-og-publikasjoner/Saker/Sak/Voteringsoversikt/?p=69815&dnid=1 (December 2, 2018).

^4^https://www.statista.com/statistics/309553/power-tool-lithium-ion-battery-market/ (December 2, 2018).

^5^https://www.umweltbundesamt.de/themen/klima-energie/erneuerbare-energien/erneuerbare-energien-in-zahlen#statusquo (December 2, 2018).

^6^www.icao.int/Newsroom/Pages/ICAO-Council-Prohibits-Lithium-Ion-Cargo-Shipments-on- Passenger-Aircraft.aspx (December 2, 2018).

^7^In the scientific literature, the term multivalent is widely used but we propose to avoid this term since it originally meant ions of multiple valence states (such as chromium). A synonym would be polyvalent. We suggest the term high-valent or highly valent. Many high-valent cations are also multivalent, but cations mostly regarded for future electrochemical energy storage devices are not multivalent (in this sense), like aluminum or magnesium.

^8^Meutzner et al. (2018a) for definitions of negative and positive electrode (mass), usually referred to as anode and cathode in a battery, which is discharged. Here, we chose to use the preceding terms as they are more general and independent of the operation mode of the battery.

^9^It has to be noted that the models used in the literature for lithium price (and resource) estimations are most probably not using lithium metal prices when calculating the lithium price evolution but prices estimated from lithium compounds (carbonates) instead (https://minerals.usgs.gov/minerals/pubs/historical-statistics/#lithium). This may introduce a bias in price estimations since when providing lithium metal, as required for high-energy lithium-metal negative electrodes in lithium-ion batteries, additional efforts in energy and cost need to be considered due to further processing.

^10^http://ec.europa.eu/growth/sectors/raw-materials/specific-interest/critical/ (December 2, 2018).

^11^http://www.world-aluminium.org/statistics/massflow/ (December 2, 2018).

^12^Lithium chloride is also used for the synthesis of aluminum metal (Mahi et al., 1986; Kipouros and Sadoway, 1998).

^13^It should be noted here that the elements of the most wide spread lead battery and the strongest growing lithium-ion battery are meant to act like mood tuners: criminal (“aggressive”) in case of lead (Sampson and Winter, 2018) and “calm” in the case of lithium (Tkatcheva et al., 2015). This shows that a technological development should also consider potential health risks of end-of-live waste.

^14^http://www.chemie.de/lexikon/Energiedichte.html (December 2, 2018).

^15^http://alionproject.eu (December 2, 2018).

^16^www.scopus.com (search term ‘aluminum-ion battery' or ‘Al-ion battery') (November 14, 2018).
